# Moving together: Increasing physical activity in older adults with an intergenerational technology-based intervention. A feasibility study

**DOI:** 10.1371/journal.pone.0301279

**Published:** 2024-03-27

**Authors:** Rachel L. Knight, Aïna Chalabaev, Kelly A. Mackintosh, Melitta A. McNarry, Joanne Hudson

**Affiliations:** 1 Applied Sports, Technology, Exercise and Medicine Research Centre, Faculty of Science and Engineering, Swansea University, Swansea, United Kingdom; 2 SENS, Univ. Grenoble Alpes, Grenoble, France; Flinders University, AUSTRALIA

## Abstract

Robust evidence supports the role of physical activity and exercise in increasing longevity, decreasing morbidity and helping older adults maintain the highest quality of life attainable. However, the majority of older adults are not sufficiently physically active and interventions are needed to change their behaviors. Familial or intergenerational contact has been positively linked to health and well-being in older adults. Therefore, this study aimed to i) establish acceptability and test the functionality and useability of a novel technology-driven intergenerational intervention targeting physical activity and age stereotypes, and ii) identify any potential issues with recruitment and retention. Four familial dyads (adult ≥ 65 and child 7–11 years) engaged with the intervention. Working collaboratively during a four-week trial, they combined daily step-counts (acquired via any activity of their choice, using PA trackers) to complete a virtual walk route using online platform *World Walking*. Thematic analysis of three post-intervention focus groups (one older adult; one child; one additional parental cohort) identified eight subthemes: Engagement; Provision of a Positive Experience; Participant Stimuli; Generated Outcomes; Operationality; Limitations; Mediators; Facilitators, and Perceptions. Participants enjoyed and successfully engaged with the intervention; when designing behaviour change interventions for older adults, flexibility within pre-established routines, individual choice, and avoiding rigidly imposed structures, is important. Strategies to challenge negative perceptions of older adults’ engagement with technology and PA should be integrated into recruitment processes.

## Introduction

Being physically active and engaging with exercise are widely acknowledged as effective strategies to increase longevity, decrease morbidity and help older adults maintain the highest quality of life (QoL) attainable [1]. However, when promoting such health behaviors with older adults, the direct transference of, albeit successful, strategies and techniques employed with younger adults, may prove ineffective [2]. Tailored, population-specific approaches are needed, and several promising concepts are actively being researched, including challenging age stereotypes, identifying and applying age-specific behavior change techniques (BCTs), and, intergenerational contact (specifically, that occurring between older adults and children, e.g., Knight et al. [3]).

Familial or intergenerational contact, either informally within the boundaries of daily life, or through structured interventions, has been positively linked to health and well-being in older adults [4, 5]. Merely having a greater contact frequency with grandchildren every month has been shown to positively impact health related QoL [4]. Prior research also suggests intergenerational contact may counteract the negative effects of age stereotypes [6, 7]. More particularly, it has been shown that intergenerational contact may protect older adults from experiencing stereotype threat [6], which refers to the stress-related responses that occur when individuals feel they are at risk of confirming negative stereotypes [8, 9]. In children, intergenerational contact may additionally assist with the prevention of stereotype embodiment (where age stereotypes are internalized into self-perceptions of aging) [10], thus potentially leading to long-term influences on perceptions of aging and health and well-being [7].

Despite the extent of interest in intergenerational interventions, their supporting evidence-base is relatively small, appearing to be, at least partially, anecdotal. Most interventional studies conducted involving children and older adults have either been focused on social cohesion or community initiatives, or, arts, education, or culturally based programs delivered in education facilities or supervized groups (for reviews, see Giraudeau and Bailly, Krzeczkowska et al., Martins et al. [11–13]). Such programs have historically been structured around the effects of volunteering on older adults, not intergenerational contact per se, with most exploring contact outside of familial relationships [14]. Where health benefits have been reviewed and positively reported [15], health-related components are often not the underpinning drivers (e.g., Fujiwara et al., Sakurai et al. [16, 17]).

Regarding physical activity (PA), it is suggested that cross-generational benefits stem from the motivation that may evolve from social support and the given potential to set and work on joint goals [18]. In the few instances where researchers have endeavoured to implement and analyse more rigorous methodologies to explore the impact of PA-driven intergenerational interventions, specifically on PA-related outcomes, they have encountered various challenges (including the ability to recruit the targeted generations and engagement with the intervention) [19] and report mixed results regarding different outcomes (e.g., no significant improvements observed in the older adults’ physical or mental health, or the children’s PA levels; the only promising finding was for upper limb strength in the older adults) [20]. Whilst intergenerational approaches to targeting health-related behaviours in older adults (i.e., PA) could indeed be promising [17, 20, 21], further research that specifically investigates the impact of intergenerational contact within PA driven interventions and collects sufficient data to expand the evidence-base, is urgently needed.

Participation in PA now often involves engaging with technology. Research indicates that multiple forms of technology-based interventions (e.g., web-based platforms, wearable monitors, smartphone apps) are deemed to be both feasible and acceptable intervention methods for middle-aged and older adults [22, 23]. However, it has been highlighted that family members often influence choices not only relating to technology use but also choice of, and participation in, physical activities [24]. The authors conclude that there is a need to design interventions that increase self-efficacy, are led by enjoyment, and foster independent active habits. Whilst technology-based interventions could provide the basis for effective strategies targeting PA, the inclusion of family members, or other intergenerational-driven approaches, could increase their appeal and ultimately success. Conversely, it has been identified that facilitating optimal intergenerational contact is, in practice, often difficult to achieve [25] as current social norms and structures mean that people predominantly interact and socialise with others of a similar age to them [26]. Exploring the use of technology, in the right context, could also provide a viable solution to this issue.

Establishing feasibility, from the perspective of all parties involved, prior to full-scale trials, or implementation, is a key development stage of the Medical Research Council (MRC) framework for complex interventions [27]. Given the issues identified in previous work (i.e., [19]), evaluating areas of uncertainty such as intervention components, recruitment and retention prevents fundamental problems in future work [28]. Therefore, the overall aims of this study were to: i) establish acceptability and test the functionality and useability of ‘Moving Together’, a novel technology-driven, familial dyadic, intergenerational intervention specifically designed to impact age stereotypes and PA levels in adults aged ≥ 65 years, and ii) identify any potential issues with recruitment and retention. Whilst the primary focus was the older adults, the potential impact and implications for their partners, children aged 7–11 years, and the views of parents who may be involved more peripherally (i.e., providing parental consent, acting as a key conduit to older adults, and facilitating child participation) were also considered.

### Theoretical framework

Despite the increasing popularity of intergenerational practices, reported developments often lack the use of theory, a strong conceptual framework, and an outcome-driven evidence-base [29, 30]. The developed intervention was underpinned by the components of Contact Theory [29], which supports the benefits of social contact between different groups. Often associated with reductions in prejudice, prior research both within and outside of laboratory settings has focused on its ability to change negative attitudes and reduce the impact or threat of stigma. Comparable outcomes have been reported whether contact is structured or unstructured [31]. However, Allport [29] suggests that we are inherently more sensitive to factors that conform to our negative perceptions and stereotypes, and that casual contact can be superficial and therefore a potential source of increased, not decreased, prejudice. Indeed, it is suggested that “true acquaintance” [29] (p. 264) is needed if stereotypes are to be effectively challenged.

**Fig 1:**
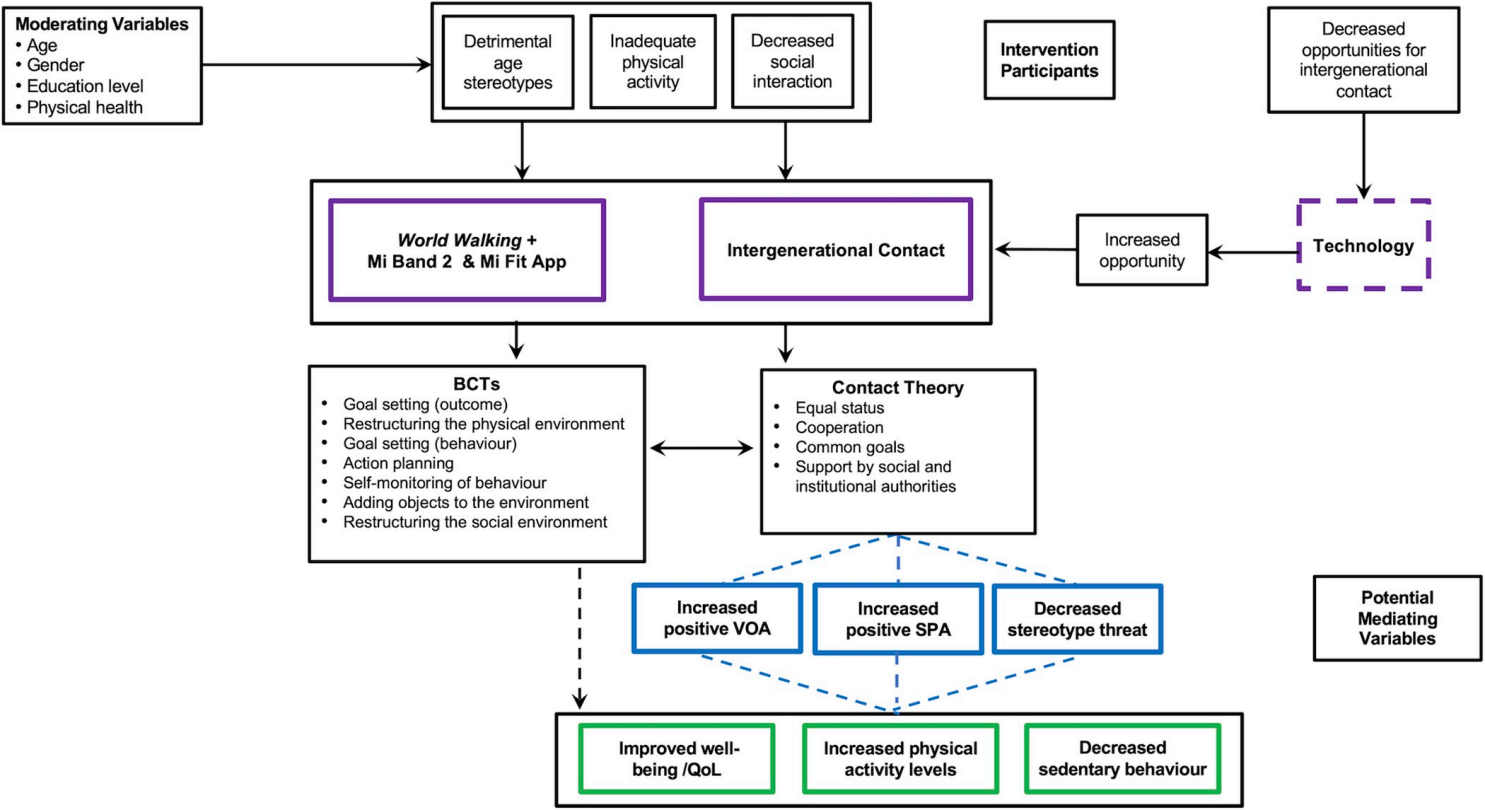
Conceptual model of technology-driven intergenerational physical activity intervention. ^a^ BCTs = behavior change techniques; QoL = quality of life; SPA = self-perceptions of aging; VOA = views-on-aging.

It was postulated that the conceptual principles of Contact Theory could potentially, either directly or indirectly through changes in attitudes and reductions in stereotype threat, be an effective method to target health-related variables in older adults. The work of Allport [29] identified four core components deemed necessary for the successful application of Contact Theory, namely, equal status, cooperation, common goals, and support from social and institutional authorities. A logic model that proposes the potential causal mechanisms by which effects on PA and other health-related outcomes (sedentary behavior and QoL) could be achieved is outlined in Fig 1.

## Methods

### Research design

This feasibility study employed qualitative research methods. A trial period of the proposed intervention of up to four weeks was qualitatively evaluated via post-participation focus groups with the children and older adults. This timeframe was deemed appropriate as four-week trial periods are regularly used in feasibility studies that aim to test the use of technology as a tool in a subsequent intervention trial, as was the case here [32, 33]. Reasons for recruitment limitations were also explored via an additional focus group held with a separately recruited cohort of parents. This data collection addition was made following realization during the recruitment process, that parents were a key link between the older adult and child, and subsequent dyad enrolment.

As this study was designed to test the feasibility of progression to a larger scale trial and is positioned within the developmental stage of the Medical Research Council (MRC) framework for complex interventions [27], no baseline or outcome measures, or exploration of intervention effectiveness was included. Moreover, as this study was conducted prior to the latest MRC guidance version publication, the study design did not include the new recommendation of including an assessment of pre-defined progression criteria. Basic participant demographics (age and gender) and metrics regarding recruitment and retention, were collated to aid the overall analysis. Ethics approval was granted by the Institutional Research Ethics Committee (approval numbers: 2018–103 and 2018-103A). Written, informed consent or assent was obtained from all participants, and the parent/guardian of each participating child. Prior to enrolment, all dyad participants were health screened by the primary researcher (RLK), using a questionnaire based on American College of Sports Medicine (ASCM) guidelines [34] to ensure there were no contradictions to participation. The study methodology, interview schedules and questionnaires were reviewed and discussed by the primary researcher (RLK) with JH, KAM, and AC, who between them have a broad range of experience across older adult and childhood PA, and stereotype-based research disciplines. The primary researcher (RLK), who implemented the intervention trial, conducted the focus groups, and carried out the primary data analysis is a registered healthcare professional with extensive clinical experience. A post-positivistic epistemological position was taken throughout this research. This reality was viewed through a critical realist lens that acknowledges that the ‘real-world’ potentially sits behind subjective and social complexities that shape a truth [35].

### Participants

Intergenerational dyad participants were recruited via purposive sampling from a single Primary School in South Wales, United Kingdom. Recruitment packs were sent to all children aged 7–11 years, with additional recruitment posters placed within school grounds and sent out via the schools’ electronic message platform. The older adult (aged ≥ 65 years) could either be a family member of the child (aged 7–11 years), or, an older adult the child had a “familiar” link with. For further inclusion/exclusion criteria see Table 1. In total, six expressions of interest were received, with four dyads consenting to participate. Two dyads were excluded because they were proposing to engage as a parent-child dyad, not a grandparent-child dyad. This, in line with previous qualitative feasibility studies [28], provided an overall sample size of eight individuals, all of whom were white British. Further demographic and participation data for each dyad are presented in Table 2.

**Table 1:** Intergenerational dyad study inclusion/exclusion criteria.

Inclusion criteria	Exclusion criteria
A dyad pairing of a child aged 7–11 years and older adult aged ≥ 65 years	No co-consenting participant within required age range
Able to write and converse in English	Unable to write and converse in fluent English
Willing to discuss their experiences in a focus group	Uncomfortable sharing experiences of study intervention with fellow participants
Access to a smart phone, computer or tablet device, and Wi-Fi/internet access	No access to a smart phone, computer, or tablet device and/or Wi-Fi access
Completed health screening questionnaire and medical clearance (where indicated)	Contraindications to participation on health questionnaire/lack of medical clearance
Available for the whole duration of the study period	Unable to cooperate with the research team for the full duration of the study

**Table 2:** Demographic and participation data of the intervention dyads.

Identifier	Older adult		Child		Intervention days completed	Pseudonyms (Older adult/Child)
	Gender	Age (years)	Gender	Age (years)		
**Dyad 1**	F	67	F	7	23	Morgan/Jesse
**Dyad 2**	M	71	F	7	28	Pat/Taylor
**Dyad 3**	F	67	M	8	28	Viv/Casey
**Dyad 4**	F	66	F	7	28	Francis/Alex

^a^ F = female; M = male

^b^ Participant availability for enrolment reduced the intervention days completed by Dyad 1

The additional focus group of parents was purposefully recruited from those who had previously contacted the researcher with queries or for further information during the recruitment process. Four individuals initially agreed to participate; however, one withdrew, resulting in three participants in the final focus group (2 females, 1 male; aged 38–47 years). Each identified at least one older adult within the age range 65–73 years, and, a child aged 8–11 years who would have been eligible to participate in the study.

### Intervention

Moving Together is a multi-faceted technology-driven intervention designed to facilitate PA in familial intergenerational dyads. Through engagement with *World Walking*, dyads combine their daily step counts (recorded via wrist-worn ‘Mi Band 2^©^*‘* activity trackers) to collaboratively complete pre-developed virtual walk routes. *World Walking* is an interactive step-count-based, open access, online platform, accessible via a computer, or additional downloaded App. The walk routes, in this instance, were along the coast of Wales in the United Kingdom. Routes are designed to include several target landmark milestones. As steps are added to the walk, the associated map route is updated to outline the percentage completed. When each milestone is reached, information of interest about the location is revealed to the user. For a breakdown of the intervention components, their associated BCTs or ‘active’ elements [36], and modes of delivery see Table 3.

**Table 3:** Components of the intervention and their associated BCTs and modes of delivery.

Intervention component	BCTs	Mode of delivery
***World Walking***	Goal setting (outcome) (1.3)	Targeted completion of a virtual walk route and attainment of interim milestones
	Restructuring the physical environment (12.1)	Access to and engagement with the platform and/or App
**Mi Band 2**^©^ **& Mi Fit App**^©^	Goal setting (behavior) (1.1) & Action planning (1.4)	Individual setting of specific daily step goal target
	Self-monitoring of behavior (2.3)	Observing and engaging with record of daily steps, providing feedback on own behavior
	Adding objects to the environment (12.5)	Provision of the wearable device, facilitation of use of the App
**Intergenerational contact**	Restructuring the social environment (12.2)	Social environment changed through the formation of the dyadic partnerships to facilitate social support and the targeted behavior

^a^ BCTs = behavior change techniques

^b^ Numbers in parentheses relate to Behaviour Change Technique Taxonomy v1 [36]

Having been facilitated to form an intergenerational partnership, supplied with the overall collaborative goal of completing the walk route within the trial period, and access to the necessary technology and resources to complete the target goal, dyads are free to set their shared daily target and generate their daily steps using any form of PA or structured exercise recordable by the Mi Band 2^©^, (including those generated through activities of daily living). The only stipulation is that *both* members of the dyad must contribute to the step totals and therefore the completion of the challenge. Activities can be undertaken either together, separately, or a combination of both, and dyads are free to establish their own preferred ways to communicate their achievements with their partner. Table 4 outlines how each parameter of Contact Theory [29] was addressed during intervention development.

**Table 4:** Contact Theory [29] intervention components.

Condition	Application within the intervention
**Equal status**	Each member of the dyad is afforded an equal role in their pursuit of their goal, using the same wearable devices, collecting the same data, over the same time periods.
**Cooperation**	The physical activity intervention requires participants to work together, not in competition.
**Common goals**	The focus of *World Walking* is the achievement of a shared common goal that relies on both dyad members’ contribution.
**Support from social and institutional authorities**	Support for the contact is provided by the relevant personnel within the place of recruitment (i.e., school headteacher) and from the parent of the child.

### Procedures

Prior to commencing the intervention, the dyads, and, a parent/guardian for each child attended a face-to-face enrolment and induction session with RLK at the Primary School, during mid-March 2019. Consent and health screening were reviewed, and basic demographic data collected. Both members of the intergenerational dyad were supplied with a “Mi Band 2^©^” activity tracker, and, assisted to download and set up the associated “Mi Fit App^©^” on the smart device of their choice. For the children, this was governed by their parent. For the older adults, access to *World Walking* was also established; the researcher provided assistance to download and set-up the App as required by each individual, which varied from providing written instructions which were followed independently, to providing practical help with the set-up. Dyads commenced intervention participation immediately following enrolment. No individual exercise prescription occurred, nor was this the intention in any subsequent planned studies. During this feasibility work, participants were only encouraged to engage with and trial the concept of the intervention. The intention of the designed intervention was to provide and stimulate a pragmatic situation replicable in the real-world. This approach, allowing for individual choice, was adopted to allow individual participation levels to be pragmatically explored, when, as per real-world situations, exercise prescription does not routinely occur.

Only the older adult could be provided with access to the *World Walking* platform due to data protection access restrictions. Therefore, the child separately recorded their step data and liaised with their co-participant for it to be added to their combined totals. The child was supplied with an A3 copy of the map/walk route, to allow them to additionally chart the collaborative progress, as per that shared with them from *World Walking*, by their partner. To allow for personal preference, each older adult was also supplied with a printed step record sheet, to record their daily steps, instead of referring to the Mi Fit App^©^. For copies of the step records, see S1 File. During weeks one to three of the study, each older adult participant and the parent/guardian of each child were briefly contacted via email to discuss any issues or concerns. The study is reported according to the Standards for Reporting Qualitative Research: A synthesis of recommendations (SRQR) checklist [37] (S2 File).

### Data collection

In total, three separate researcher-led focus groups were held, one for the child dyad participants, one for the older adult dyad participants, and one for the additionally recruited non-participant parents. The focus groups with the dyad participants were designed to obtain information about participants’ experiences of using and interacting with the fitness tracker and technology platform, and their overall experiences of collaborating and working intergenerationally, to inform future recommendations for implementation within a larger scale trial. Regarding the older adults, the participants’ opinions concerning the impact of the intergenerational collaboration on their views-on-aging, and age stereotypes in general were also explored. The additional focus group undertaken with the recruited non-participant parents discussed participant recruitment issues/limitations, the overall intervention concept, opinions, and potential solutions. For the outline interview schedules for each group, see S3 File.

All focus groups, undertaken following the completion of the intervention trial period at the recruitment Primary School, were conducted by the primary researcher RLK, in a non-direct, neutral manner [38]. The sessions, ranging in duration from 20 to 75 minutes, were audio and video recorded (Phillips Digital Voice Recorder; Sony HandyCam), transcribed verbatim by RLK, and anonymized. Where the focus group involved children, due to the room location, to comply with child safety requirements, a teaching assistant, identified by the school was also in attendance.

### Data analysis

Focus group analysis was undertaken drawing on the thematic analysis process outlined by Braun and Clarke [39, 40]. Using this iterative structured approach, the coding process was deductively driven by a pre-defined, study aim-specific, individually constructed thematic framework. Under the headings: acceptability; functionality; useability, and, recruitment and retention, codes were allowed to emerge inductively. Only the semantic meaning of the data, as presented by the participants, was explored [41].

All coding was initially conducted manually by RLK, with subsequent categorization, organization, re-checking, and refinement carried out within Microsoft Word (Office 365^©^). Following the initial coding process, a second researcher (JH) blindly cross-matched 10% of the data extracted against the generated codes to ensure consistency in approach and appropriate data coding. From the 10% cross-matched, eight discrepancies were discussed and reviewed back to the original data set until agreement was reached, with the remainder of the initial coding re-reviewed, as required. Codes and sub-themes were challenged and checked back against the original transcripts to ensure data fit, removed and/or rearranged to ensure accurate data representation, and renamed accordingly. These processes were, for transparency, credibility, quality control and rigor [42], completed in collaboration with a ‘critical friend’ (JH). Examples from each stage of the process are provided within the coding audit trail (see S4 File).

### Reflexivity for trustworthiness

It is pertinent to note that the research was undertaken in a location where the primary researcher (RLK) was known to some of the participants and the wider Primary School community. Consideration therefore needs to be given to the way in which this might have affected interaction and responses. This particularly related to the non-participant parental group, who all had a priori knowledge of the primary researcher (RLK) and their professional background as a healthcare professional. At all times during data collection impartiality was strived for. As the aim of the research was to establish feasibility, the common codes and sub-themes triangulated from different perspectives across all data sets were afforded equal importance [43].

## Results

**Fig 2:**
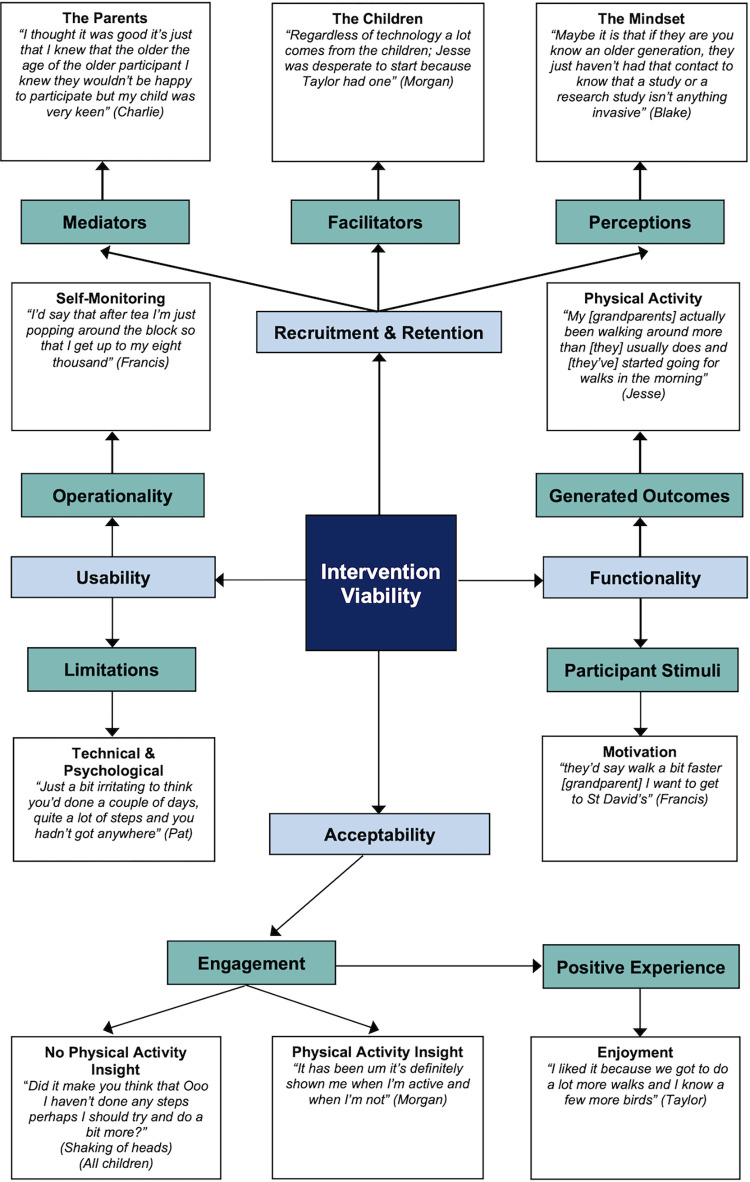
Pen profile of core feasibility themes related to participant engagement with and views of a collaborative intergenerational technology-driven intervention.

Through the deductive application of the four pre-defined over-arching framework themes that targeted the parameters of the study objectives, eight core themes were identified: Engagement; Provision of a Positive Experience; Participant Stimuli; Generated Outcomes; Operationality; Limitations; Mediators; Facilitators, and Perceptions. These are presented visually with associated quotations using a pen profile [44] (see Fig 2). The pseudonyms Morgan, Francis, Pat, and Viv relate to the older adults, focus group 1 (FG1:OA); Jesse, Alex, Casey, and Taylor to the children, and focus group 2 (FG2:CH); Blake, Charlie, and Sam to the non-participant parents, focus group 3 (FG3:P). For the older adults and children, D1-D4 indicates their associated dyadic pairing (see Table 2).

### Acceptability

#### Theme 1: Engagement

Overall acceptability of the intervention was supported by the level of engagement with the core concepts and components. With regards to the technology, whilst the primary focus was interaction with the step count features, and the ability of these features to facilitate the use of *World Walking*, participants also reported accessing additional features of the watch and App: *“I liked it because you got to count your steps and you knew your heartbeat” (Alex; FG2*:*CH*:*D4)*.

*Pat*: *“Um not just for the steps but for the sleep patterns as well*, *we were having a chat about that earlier on um I found that quite fascinating I’m tempted to buy one to keep it going*” *(FG1*:*OA*:*D2)*

Both groups of participants, and the parents of the children, thought that *World Walking* was interesting: “*I think the map was the main thing it was the interest really in seeing how far we were getting it was good for us and good for the children as well” (Pat; FG1*:*OA*:*D2)*, and, that the children in particular enjoyed charting their progress: *“Ooo Ooo I liked it because I keep getting putting stickers on my umm map” (Casey; FG2*:*CH*:*D3)*. However, frequency of engagement varied. Where some checked their steps and progress every day, others, more specifically the parents assisting the children, synced the watch and updated their progress three to four times per week.

An important observation is the impact that engagement had on the participants’ insight into their activity levels. For the older adults, engagement appeared to have a constructive effect. Participants demonstrated a change in their awareness of how active they were, when, and, what factors positively (i.e., increased awareness of PA opportunities) and negatively (i.e., looking after grandchildren) affect their behavior.

*Morgan*: *“It has been um it’s definitely shown me when I’m active and when I’m not um (*.*) I look after my [grandchild] a little one two days a week sometimes three and* *then* *my steps are really down because I’ve got*” *(FG1*:*OA*:*D1)*

*Viv*: *“I have found myself more aware of exercise um (*.*) like I walk around the bathroom cleaning my teeth now ((laughter)) and when vacuuming the carpet instead of standing on the spot and going like this ((demonstrates)) I go striding down the hallway and striding back up again ((laughter)) so it’s made me more conscious then*” *(FG1*:*OA*:*D3)*

However, for the children themselves, such an awareness of this association, did not appear, at least knowingly, to be as consistent:

*Casey*: *“Well I did leave it somewhere*, *but I tried to get more steps up*” *(FG2*:*CH*:*D3)*

*Jesse*: *“Uh but … I think we should do it again because it might actually help people be encouraged a bit more to do a bit more walking and get fit more*” *(FG2*:*CH*:*D1)*

#### Theme 2: Provision of a positive experience

Taking part in the trial and engaging with the intervention process was considered a positive and enjoyable experience by all involved: *“Yeah and I’ve quite enjoyed doing that and it made me feel fitter and better for doing it” (Francis; FG1*:*OA*:*D4)*, *“I didn’t dislike anything really” (Viv; FG1*:*OA*:*D3)*. All of the dyads were composed of grandparents and grandchildren. The older adults specifically, recognized not only an influence on their fitness levels, but, also on their relationships and contact with their grandchildren. The opportunity afforded to them to consolidate and explore this intergenerational relationship was deemed to be a good thing.

*Pat*: *“I thought it was good because um ((pause)) I think you have a different relationship with your grandchildren to your children to some extent and so it was although we see a lot of ours it was just a nice thing to do*” *(FG1*:*OA*:*D2)*

As well as the short-term intervention specific gains, wider benefits, and, the potential for longer term engagement were identified. Some participants found the watch *“addictive”*, whilst others were considering purchasing their own.

*Alex*: *“I’m actually getting one of my own which is waterproof*” *(FG2*:*CH*:*D4)*

The potential to instigate wider reaching gains and changes in behavior not just directly for the dyad members, but also indirectly for other family members was also discussed. Children reported being more active with other family members: *“I liked it because um I because I we got to do a lot more walks and now*, *I get to know a few more birds because we’ve gone out for lots more walks” (Taylor; FG2*:*CH*:*D2)*, and, plans had been made to form larger familial teams to complete longer challenges in the future: *“when this trial is over we are going to carry on the four of us no five*…… *and target walking to the moon” (Morgan; FG1*:*OA*:*D1)*.

### Functionality

#### Theme 3: Participant stimuli

From the basic principle of knowing they were measuring activity, to the satisfaction gained from observing higher daily step count levels, and, the encouragement received from their co-participant: *“they’d say walk a bit faster [grandparent] I want to get to St David’s” (Francis; FG1*:*OA*:*D4)*, the motivation provided was repeatedly drawn upon.

*Morgan*: *“It’s good in that way in that it’s made me more aware of it (*.*) it made me more conscious of it and made me think and basically as you say rather than sit down and think aw I’ll do something later I’ll do it now you know so it does it’s a good*” *(FG1*:*OA*:*D1)*

*Viv*: *“Motivator*” *(FG1*:*OA*:*D3)*

*Morgan*: *“It is a good motivator there’s no doubt about that*, *for me anyway*” *(FG1*:*OA*:*D1)*

*World Walking* and the underpinning principles of the intervention trialed are based on the premise that collaboration, rather than competition, is a key driver for success. With the dyads’ accumulated step counts being used to complete the walk, the notion of “working together” was received positively. For example, *“If I was doing it on my own*, *I wouldn’t have got very far but when we when me and my [grandparent] were working as a team we got quite far” (Taylor; FG2*:*CH*:*D2)*, *and “Jesse had the map and of course [they were] following it as well and saying*, *‘come on [grandparent] you need to do more’*… .*” (Morgan; FG1*:*OA*:*D1)*. However, despite not being targeted within the intervention design process, it was clear that the participants also enjoyed competing with each other: *“I like it because I keep beating my [grandparent]” (Casey; FG2*:*CH*:*D3)*. It is pertinent to note that this competition was not viewed negatively: *“when we say was competitive it was just a bit of fun isn’t it really” (Pat; FG1*:*OA*:*D2)*, and, in fact it provided an additional source of motivation.

*Francis*: *“It does make you more competitive I think I mean obviously if I spoke to Alex on the phone and they’d say*, *‘how many steps you done*?*’ and if I’d done more than them they’d tell me ‘talk to [parent]’*” *(FG1*:*OA*:*D4)*

Where endeavoring to achieve the collaborative goal was important, also important was individualization. It appeared that for the older adults particularly, setting goals that are achievable and realistic, could be of paramount importance.

*Morgan*: *“I didn’t realize that you need to do you know 10*,*000 steps is not far off 5 miles a day for me which is quite a lot to do you know when they say you should be doing 10*,*000 steps a day there’s no way I could do that I don’t think*” *(FG1*:*OA*:*D1)*

*Viv*: *“Yes if you’d set ours at ten thousand steps a day*, *I don’t think* ….” *(FG1*:*OA*:*D3)*

*Pat*: *“Yeah we wouldn’t have bothered*” *(FG1*:*OA*:*D2)*

Indeed, it was suggested that failing to acknowledge these factors could stimulate the formation of detrimental barriers to success: *“I think the important thing is if you do think about setting targets for people is*, *they have got to be achievable otherwise you get that demotivating factor coming in” (Pat*: *FG1*:*OA*:*D2)*.

#### Theme 4: Generated outcomes

Participation in the trial period was generally felt to have had a positive impact on the primary targeted behavior, PA. Some participants saw it as an opportunity to make time: *“I’ve always enjoyed doing it when I’ve had the time but what this has made me do is make time” (Morgan; FG1*: *OA*: *D1)*. For others, it offered a way to find methods of incorporating more activity and make active choices within existing daily routines.

*Viv*: *“sometimes if it was a nice day*, *I would walk the long way around I’d come right up to the [Club] and come around to the school that way which backfired one morning because they had locked the gate couldn’t get in there was a crowd there so I just sort of circled round to clock up some steps*” *(FG1*:*OA*:*D3)*

Discovering that they actually enjoyed engaging with the intervention and finding the time to be more active surprised the older adults; whilst some of the children noticed changes in the behavior of their co-participants: *“Well my [grandparents] actually been walking around more than [they] usually does and [they’ve] started going for walks in the morning around where [they live]” (Jesse; FG2*:*CH*:*D1)*.

Not substantially altered, was the level of contact between the dyad members. Most co-participants were already in regular contact with each other at least once a week: *“I see them nearly every day anyway apart from Saturdays and Sundays so just saw them the same” (Viv; FG1*:*OA*:*D3)*. Although, some extra contact via phone calls was instigated by a few of the children, and, happily welcomed and appreciated by the older adults: *“Taylor rang me and [they don’t] normally ring me and was quite chatty on the phone talking about this and it was quite nice from that point of view*, *but we do see regularly anyway” (Pat; FG1*:*OA*:*D2)*.

### Usability

#### Theme 5: Operationality

The multi-component nature of the intervention inherently left it open and susceptible to user/interface issues, but overall, this was not the case, with the watches being deemed “*very easy*” *(Alex; FG2*:*CH*:*D4)* to use. Only one participant identified a potential synchronization issue between the watch and the App; however, this was counteracted by changing to manually noting daily step counts at the end of each day until the issue could be resolved. It is apparent, that in situations where technology-driven approaches are used, clear explanations supported by the provision of concise supporting secondary guidance are imperative.

*Pat*: *“I found it a bit baffling the day we came in and you explained it all to us*, *but once I*, *you know*, *you explained it well*, *we set things up together*, *I went home and read through the guidelines and then it was okay after that*, *I didn’t have any problems at all to be honest*” *(FG1*:*OA*:*D2)*.

All participants engaged with the technology to self-monitor their daily step progress: *“I got I got to 12*,*000 the other day” (Alex; FG2*:*CH*:*D4)*. For the children, again, this did not appear to knowingly translate into purposeful changes or increases to their PA engagement. For the older adults, a more direct association is plausible: *“If you have a day when you don’t do much like when you’re looking after one of the grandchildren you think oh tomorrow*, *I’ve gotta do some extra” (Pat; FG1*:*OA*:*D2)*, *“Yeah I think oh I’ll just pop-up town now you know I won’t get that tomorrow I’ll get that now and then I’ll get up my steps today” (Francis; FG1*:*OA*:*D4)*.

There was an observed need for parental involvement for a number of reasons. Whilst some of the dyads liaised directly with each other to transfer data and discuss their process: *“I’d phone up and tell you can stick a sticker on Bangor” (Viv; FG1*:*OA*:*D3)*, others relied on parental facilitation: *“My [person]-in-law [they were] on the phone most nights you know saying what are your steps” (Morgan; FG1*:*OA*:*D1)*. The children also needed reminding to record their steps and put their watches back on when removed, and, help to chart their progress on their maps. However, the amount of time needed was minimal, with an average parental time of five and half minutes per day spent assisting their child.

#### Theme 6: Limitations

Despite the overall positive perceptions of the intervention concept, limitations of both a technological and psychological nature were uncovered: *“I thought it would motivate me to get the bike out the back of the garage and go on a cycle ride with Casey you see but no it was all about walking” (Viv; FG1*:*OA*:*D3*). Disappointment that certain activities did not count towards daily step totals, for instance cycling, swimming, team sports requiring watch removal, was apparent: *“Jesse often said oh I did this*, *and I did that*, *but I had to take my watch off” (Morgan; FG1*:*OA*:*D1)*.

Another negative factor raised was the distance between the milestones on *World Walking*. It was felt to be disheartening and *“just a bit irritating to think you’d done a couple of days*, *quite a lot of steps and you hadn’t got anywhere” (Pat; FG1*:*OA*:*D2) and* seemed to be “*stuck*” for days at a certain location: *“I’d check it every night and go what I haven’t moved I’m still in Portmerion” (Viv; FG1*:*OA*:*D3)*. This was consequently thought to be demotivating. Uncertainty regarding a feature of *World Walking* that allocates medals was also mentioned.

The issue of compliance raised interesting points. The necessity for the removal of the watch by children for participation in water-based, and, certain other, activities leading to them forgetting to put it back on, was to a degree, not unexpected: *“I’ve left this in the toilet (laughter)*, *and I left it in the (bed) ‘cos it’s not waterproof that’s why I took it off” (Casey; FG2*:*CH*:*D3)*. However, prior consideration had not been given to the issue of parents intermittently wearing the watches for the children: *“My [parent] wears mine so [they] actually does the steps for me for a bit” (Jesse; FG2*:*CH*:*D1)*, *“I go swimming for an hour*, *so I got my [parent] to wear the watch” (Alex; FG2*:*CH*:*D4)*.

### Recruitment and retention

#### Theme 7: Facilitators

Engaging individuals with research, particularly older adults, can be difficult. Three ways to potentially enhance recruitment levels were identified. First, it was thought that the strategy implemented, targeting recruitment via the children, was the right approach, and, if anything a stronger emphasis on this should be employed.

*Morgan*: *“Right I’d have thought a lot of it would’ve come from the children um I’m in it because Jesse wanted to do it you know if she had come home and said oh*, *they’ve got this thing*, *or this letter and I don’t really wanna do it Mum and I don’t really wanna do it Dad then that would be the end of it*” *(FG1*:*OA*:*D1)*

Indeed, not only could it be the reason that older adults choose to participate, but it could also have a domino effect on stimulating interest amongst other children: *“regardless of technology a lot comes from the children; Jesse was desperate to start because Taylor had one” (Morgan; FG1*:*OA*:*D1)*, *“Once one is doing it*, *it makes other ones want to get involved” (Francis; FG1*:*OA*:*D4)*.

Second, alternative options to the familial dyad were discussed. Thoughts were mixed on changing the structure to include the ‘middle generation’. Where some felt it could work: *“I don’t know why it wouldn’t work obviously every family is different” (Pat; FG1*:*OA*:*D2)*, others felt it could change the unique dynamic of the dyad: *“because it’s Jesse and me and we’re competing then I think that’s a better motivator if that’s what you’re after is motivation” (Morgan; FG1*:*OA*:*D1)*. Regardless of the additional technological support it could add, whether this would actually encourage already skeptical older adults to become involved was debatable. Finally, it was suggested that exploring the use of incentives to boost interest and uptake, a method that often affords success in other situations should be considered: *“a couple of um surveys I’ve done you get ten pounds for them each” (Sam; FG3*:*P)*.

#### Theme 8: Mediators

Where some factors could directly facilitate uptake and participation rates, others could mediate strategy effectiveness. Often the first point of contact, the provision of sufficient study information to all concerned parties, is crucial. Where the volume is too large, it could be *“information overload for some elderly people” (Blake; FG3*:*P)*, and that could ultimately disengage people: “*it was a big folder wasn’t it bit scary wasn’t it you know what I mean” (Sam; FG3*:*P)*. This provides a dilemma for researchers undertaking multi-participant work. Getting this wrong may hinder progress.

Despite the overall focus of the intervention concept being to encourage people to move more, through reducing sedentary time or increasing PA, these were not identifiable as reasons for engagement. The level of initial and sustainable interest in these and other core elements, could inherently be limitations. Indeed, some individuals may have a distinct lack of interest in being active: *“[They] don’t like doing anything [they’re] a typical teenager even though [they’re] eleven but [they are] a teenager [they’ll] sit there watching TV or read*, *read*, *read” (Sam; FG3*:*P)*. Others, particularly older adults, whilst potentially having the capabilities to use different technologies, either may not have any interest in using it, choose not to, or, are uncertain of the terminology that surrounds it: “*My [in-law] was instantly um ‘what I’ve got to wear something*?*’ and we were like ‘yeah it’s a watch’…*. *and ‘but I wear a watch already’ I was like ‘yes’ …*. *‘what it tracks me*?*’*… .*” (Blake; FG3*:*P)*. However, the opportunity afforded to seemingly help someone else, appeared to be important: *“I hoped that they’d see that it’s not just helping them it’s helping their grandchild” (Blake; FG3*:*P)*.

*“I felt kind of happy because sometimes some days when we didn’t go out for a walk I’d only do something like 2*,*000 when my [grandparent] would be out doing lots of steps so [they] kinda helped me when [they] didn’t do lots of steps I did when I didn’t do lots of steps [they] helped me do lots of steps*” *(Taylor; FG2*:*CH*:*D2)*

Even when engagement has been achieved and acceptability established, it is apparent that there is an underlying risk that adherence and long-term participation could be hindered by the potential novelty factor. Within the relatively short time period that the intervention was trialed, activity levels were felt to have “*waned*” from those initially achieved: *“The first couple of days I walked down to town instead of taking the car but the novelty of that soon wore off” (Pat; FG1*:*OA*:*D2)*, with the children additionally sometimes showing more interest in other aesthetical features and components of the technology: *“I liked it because I could see the time” (Alex; FG2*:*CH*:*D4)*.

The final mediator recognized is one that has the potential to detrimentally impact recruitment within any intergenerational research where the target populations include older adults and children; the dynamics present with families. Through their own pre-established opinions and subsequent actions, parents may consciously or subconsciously, impede: *“I thought it was good it’s just that I knew that the older the age of the older participant I knew they wouldn’t be happy to participate but my child was very keen” (Charlie; FG3*:*P)*, or, facilitate recruitment: *“They see obviously me and [my spouse] wear them and you just yeah [they] couldn’t wait to wear one” (Blake; FG3*:*P)*. It was also believed that the prevalent pattern of an increasingly smaller age gap between generations within western societies would limit the ability to form dyads constructed of an older adult and child within the required age ranges: *“people having children earlier in life you’re not going to get a grandparent in that right bracket” (Pat; FG1*:*OA*:*D2)*.

#### Theme 9: Perceptions

It is clear that perceptions have a complex and varied role within the research recruitment process. The intervention was well perceived: “*I thought it was lovely I had children who wanted to take part and were keen to take part” (Blake; FG3*:*P)*. Nevertheless, this did not equate to the desired recruitment numbers. Perceptions of the technology, the time required to engage with being more active, and, the research process in general, were all described as limitations: *“Maybe it is that if they are you know an older generation*, *they just haven’t had that contact to know that a study or a research study isn’t anything invasive” (Blake; FG3*:*P)*.

Unfortunately, a number of negative perceptions regarding aging and the aging process were weaved throughout the focus group discussions. Some of these views were presented as likely self-perceptions:

*“They’re often in the mindset like with mine well my [parent] goes ‘well I’m seventy-two I’m not going to lose weight now am I I’m not going to’ and I’m like well you could actually you could get fitter you could move more but [they are] you know ‘I’ve had a good life’ and ‘I’m seventy something’ ‘I’m gonna keep as I’m going*’……” *(Blake; FG3*:*P)*

Moreover, older adults were viewed by others as being too set in their ways to embrace a new challenge that would potentially interfere with their daily routines: *“I think it’s an age thing as well they’re all set in their own ways of what they will do at certain times and they’ve got routines and I think that is what is the main problem” (Charlie; FG3*:*P)*. Technophobia and the ability of older adults to use the required technology was also questioned: *“I would actually wonder about if it’s the technology that put older people off a little bit because not everyone over sixty-five is conversant with modern technology” (Viv; FG1*:*OA*:*D3)*.

## Discussion

### Intergenerational physical activity: A positive approach?

All participants successfully engaged with the intervention for the whole duration of the trial period indicating acceptability, and, the provision of a potential platform to generate positive behavioral changes and health outcomes. Participants signaled that they enjoyed taking part. Enjoyment, particularly when considering or undertaking more PA has been deemed an important motivational factor for both older adults [45, 46] and children [47]. However, it is pertinent to note that the reasons for impact, and therefore the underpinning mechanisms at work, may be different for dyad members within each targeted age range, in this instance older adults aged ≥ 65 years and children aged 7–11 years.

It was observed that where the older adults appeared to draw direct and explicit associations between daily monitored step counts, self-determined goal progress and success, for children, the effects could be more subtle and implicit. In this situation, the children’s benefits appear to have arisen from their desire to “beat” their co-participant and complete the walk. The application of behavior change models to one population age group, just because they have demonstrated success with another, has already been questioned [2], therefore, the different mechanisms of change and effect, are not surprising. Exploration of the potential impact of different variations of interventions of this nature, to optimize the benefits to all age ranges, could be warranted.

Intergenerational contact provides both children and older adults with the opportunity for generativity, a concept identified as a potentially important driving force in successful partnerships and outcomes [16, 48], and, within this study, a recruitment facilitator. Whilst classically presented as an opportunity to guide and help the next generation [49], through its ability to determine self-worth in later life [50], it appears that in pre-pubescent years, children may also be able to identify and attach comparable benefit to the perceived ability to help others.

### Is the stereotype cliché getting old?

Intergenerational contact has been proposed as a way to target the detrimental effects and limit the impact of negative stereotypes of aging across generations [51–53]. The work of Abrams et al. [51] reported that the effects of stereotype threat on older adults aged 59–89 years, were notably suppressed when prior contact with young people had been more positive. Comparison with outgroup members significantly impaired cognitive performance in individuals who experienced less contact, with those who had experienced higher levels of contact relatively unaffected.

Levy et al. [54] found that both implicit and explicit stereotype manipulations led to increased physical function in older adults, however implicit strategies achieved effects that were 30% greater. Intergenerational contact could be viewed as an implicit stereotype manipulation, where, through applying the theoretical components of Contact Theory [29], self-perceptions of ageing, and views-on-ageing in older adults, and attitudes towards ageing in children, are subtly challenged in line with the principles of stereotype embodiment theory [10]. Indeed, although preliminary, the findings of this study indicate that this could be a viable proposition. Alternatively, positive changes could be the result of the construction of an optimum situation and environment, that is context-specific, and again, underpinned by the parameters of Contact Theory [29], leading to reductions in perceived stereotype threat [9]. Ironically, within this feasibility study, negative stereotypes of aging, both self-perceived and views-on-aging, particularly relating to the abilities of older adults to engage with technology and be physically active, were evident. Additional strategies to challenge such stereotypes may need to be an integral part of recruitment processes.

### Family: Friend or foe?

Where associations have been made between social support levels, and physical inactivity in older adults [55, 56], the role of the family unit, its structural make-up, and hierarchy within in it, is undoubtedly complex. Important observations were raised regarding the targeted age range inclusion criteria, and the impact this could have on the availability (i.e., due to the generational age gap), and accessibility (i.e., due to the pre-conceived views of the wider family), of the corresponding generations. Allowing ‘younger’ older adults to participate was a suggested solution. Whilst this would potentially change the boundaries of any conclusions that could be drawn, it has been suggested that age stereotypes may actually become less threatening with advancing age [57], and, that salience to stereotype threat in particular, is indeed greater, during the transition into older adulthood [58]. Therefore, intergenerational interventions that target ‘early’ older adulthood could be more effective.

When designing and ultimately endeavoring to implement intergenerational interventions or programs, how members of the wider family will view and engage with the concept also warrants consideration. Strategies to challenge their beliefs and pre-conceived opinions may be essential. Particularly with familial older adults and children, the parental ‘gatekeeper’ who may end up mediating participation, could, albeit unintentionally, considerably help or hinder success [59]. However, the extent of any impact may not always be initially apparent.

### How do we solve the problem of recruitment?

Perhaps the biggest challenge facing intergenerational research, particularly where the target populations are older adults and children, is how to effectively recruit sufficient participant numbers. Addressing this issue is of paramount importance, as at present, the evidence-base surrounding this concept and its ability to positively affect health outcomes in older adults is at best, anecdotal. In line with the encountered limitations, prior studies have experienced similar issues. For instance, the *iStep* project initially aimed to explore the effects of a pedometer-based intergenerational social innovation on obesity levels in older adults through the formation of grandparent/grandchild partnerships. Unsuccessful recruitment led to the formation of pupil/teacher, and pupil/parent partnerships instead [19, 60]. Regardless of the level of potential afforded to an intervention concept or behavioral change strategy, or, how accurately it is constructed, a failure to recruit limits the ability to explore engagement, the magnitude of any observed change, and, the wider transferability of results [61].

There is an apparent need to address the divide between how the research process is presented and subsequently viewed. Targeted, population specific, innovative recruitment strategies need to be devised that evoke interest, demonstrate a positive benefit to burden ratio, and, where necessary, subtly challenge the perceptions and opinions of not only potential participants, but also their wider circle of often influential family and friends [59]. Additionally, specific to interventions utilizing technology, consideration of how terminology is used, and, the connotations that could arise from different interpretations of seemingly standardized wording, for example, ‘activity tracker’ is needed.

Reporting the lessons learnt from recruiting 777 older adult participants, aged ≥ 65 years with a high risk of mobility disability into a 12-month multicenter RCT, Withall et al. [62] adopted a variety of recruitment strategies. The most effective strategy appeared to be mail invites via General Practitioners. Face-to-face recruitment via liaison with and presentations at third-sector organizations (i.e., shelter housing) provided minimal uptake. However, noteworthy is their recommendation that to gain a representative sample, and therefore increase transferability, such relationship building methods may still be essential with minority groups.

### What are the key considerations for future work?

For older adults, when designing behavior change interventions, whether intergenerational or not, the findings of this study suggest it may be pertinent to consider whether they allow for flexibility within pre-established routines and individual choice. They also need to account for a potential lack of interest in and engagement with, rigidly imposed structures, and enable ‘activity’ to occur as a by-product of participation in other activities [63]. It is however noted that the effectiveness of self-regulatory techniques with older adults is questionable [2, 64]. Within this study, attainable achievement was also perceived as being important, where the distance between some of the milestones within *World Walking* was deemed to be too far, motivation waned.

Another key ‘gripe’, that could have been a contributory factor to the issue that arose whereby some parents were wearing their children’s watches, was the inability to record steps for other sports and activities undertaken. Consideration needs to be given to how this issue can be addressed. One possibility could be the provision of activity-to-step conversion charts, thus allowing the accumulation and addition of equivalent step data. Less despondence from the children could remove the need for parents to feel compelled to help. Some purposefully constructed, study specific, web-platforms have utilized more sophisticated built-in step calculators [65], however, within real-world research, options are restricted.

### Strengths and limitations

Within this study, 75% of focus group participants and 100% of parental responses were female. Positively, each focus group did have one male representative. This is important as it has been noted that gender disparities within such research are common [15], and, that males and females could have different outcome responses to intergenerational interventions and activities [53]. Criticism may also be drawn towards the small convenience sample obtained from only one local school and hence the limited scope for transferability of the results. However, given the identified recruitment complexities, and failure of other studies to recruit any of their targeted sample [19, 60], this work still provides some valuable insight. Additionally, the strategies employed within the interview schedules with the children appeared to be effective. Given the young age of the participants, and their developing linguistic ability [66], the use of monosyllabic responses was minimal, and the volume of data obtained deemed sufficient. It is, however, pertinent to note, that for some of the sub-themes (i.e., engagement) the supporting data is more heavily reflective of adult, rather than child, perspectives. In line with the study aims, the interview schedule questions focused on the novel technology aspects, rather than the context-specific behavior change strategies and their implementation. The inclusion of questions on these parameters could have broadened the depth of knowledge generated. Finally, the dyads only experienced a relatively short trial period. It is therefore difficult to truly understand the implications of any novelty factor or longer-term adherence issues. Whilst the developed intervention was underpinned by the components of Contact Theory [29], future large-scale interventions are required to ascertain *how* Contact Theory relates to the findings.

It is pertinent to note that the primary researcher was a parent at the participating school, and, the only focus group moderator. Whilst professional and ethical boundaries were always observed, an impact on recruitment, and, the subsequent results, albeit positive or negative, cannot be ruled out. To increase rigor and trustworthiness, within the focus groups, outline interview question schedules were specified a priori, and, within the data analysis, identified codes and themes were reflected on and challenged by a critical friend to ensure that the personality, experiences, and, beliefs of the researcher, and, goals of the research did not influence the analysis and reporting.

## Conclusion

This study provides a limited yet encouraging and constructive insight into the effects of an innovative approach to targeting PA engagement and stereotypes of aging, and considerations for future work. Broaching physical inactivity and sedentarism through technology-driven intergenerational contact provides a viable option for further controlled exploration. Where motivational drivers and the level of direct impact may differ between dyad members this should not be viewed negatively, especially if interventions are designed with a primary emphasis on the health outcomes of one half of the dyad (i.e., the older adults). It may need to be accepted that, whilst secondary gains are still there to be made (i.e., for the children), they may not be as significant.

## Supporting information

S1 File Step record charts. (PDF)

S2 File Standards for Reporting Qualitative Research: A synthesis of recommendations (SRQR) checklist. (PDF)

S3 File Interview schedules. (PDF)

S4 File Coding audit trail. (PDF)
