# Supplementary material for: Moving together: Increasing physical activity in older adults with an intergenerational technology-based intervention. A feasibility study
Source: PLoS One. 2024 Mar 27;19(3):e0301279. doi: 10.1371/journal.pone.0301279 (PMC10971589; doi:10.1371/journal.pone.0301279)
Supplement: S1 File — (PDF) [file pone.0301279.s001.pdf]

S1 File

Step Record Charts

	Daily Goal	Monday	Tuesday	Wednesday	Thursday	Friday	Saturday	Sunday
Week 1								
Week 2								
Week 3								
Week 4								

	Week 1	Week 2	Week 3	Week 4
Comments				

	Monday	Tuesday	Wednesday	Thursday	Friday	Saturday	Sunday
WEEK 1 Write in your number of steps							

Colour the footprints or add a sticker to mark off each time your steps are added to World Walking

	Monday	Tuesday	Wednesday	Thursday	Friday	Saturday	Sunday
WEEK 2 Write in your number of steps							

Colour the footprints or add a sticker to mark off each time your steps are added to World Walking
