# Supplementary material for: Moving together: Increasing physical activity in older adults with an intergenerational technology-based intervention. A feasibility study
Source: PLoS One. 2024 Mar 27;19(3):e0301279. doi: 10.1371/journal.pone.0301279 (PMC10971589; doi:10.1371/journal.pone.0301279)
Supplement: S3 File — (PDF) [file pone.0301279.s003.pdf]

S3 File

Focus Group Topic Guide, Older Adults

Introduction

Participants will be reminded that:

- Whilst the session will be recorded, and the researcher will take notes as needed, the recordings will be destroyed following full data analysis.
- Everything they discuss will remain anonymous, they can contribute when they choose to do so, and, that there are no right or wrong answers, different people might have different views on the intervention, and we are interested in finding out everyone's experience of the intervention.

They will then be given an opportunity to ask questions before the focus group begins.

Main Questions

- What did you think of taking part in the intervention?
  - Did you enjoy it, dislike it, like it, find it helpful or unhelpful in any way?
- What did you think about having to pair up with the child?
  - Do you think it made any difference? Did you like it, dislike it? How did it make you feel?
- Did taking part encourage you to be more active?
- How did taking part make you feel and think about being an older person?
- How did you find using the technology practically?
  - Do you feel that you had the right skills to use technology?
  - Did you have any issues accessing the web page/App relating to Wi-Fi/internet access?
  - Think about the watch - Was it easy to use? Any difficulties?
  - Think about the website/App – Was it easy to use? Any difficulties?
- Would they use/recommend using this type of intervention in future?
  - Why? Why not? What alternatives would you suggest?

Summary

- Reminder that if participants want to withdraw their data from the study, they just need to contact the main Researcher without offering a reason.
- Participants thanked for their time and given the opportunity to ask questions about the study within the group or privately.

Focus Group Topic Guide, Children

Introduction

Participants will be reminded that:

- Whilst the session will be recorded, and the researcher will write notes as needed, the recordings will be deleted once they have been used.
- Everything they discuss will remain confidential, they can answer when they want to, there are no right or wrong answers, different people might give different answers, but we are interested in finding out everyone's experience and opinion.

They will then be given an opportunity to ask questions before the focus group begins.

Main Questions

- Have you liked taking part in the activity over the last few weeks?

    If yes.... Could you tell me a little bit more? Did you enjoy it? What did you enjoy?

    If no..... Could you tell me a little bit more? What did you dislike?
- Do you think taking part made you be more active?

    What did you find helpful/unhelpful?
- How did you find using the watch and having to wear it?

    Was it easy to use? Any difficulties?
- Did you enjoy using your map?

  What did you do? How did you find out how far you had gone?
- What did you think about having to pair up with an adult/your grandparent?

    Do you think it made any difference? Did you like it, dislike it? How did it make you feel?
- What did you think about the adult taking part?
- Would you like to do something like this again?

    Why/why not? What could we do to make it better?

Summary

- Reminder that if they decide they don't want what they have said included in the study, they just need to get their parent to contact the main Researcher.
- Participants thanked for taking part and given the opportunity to ask questions.

Focus Group Topic Guide, Non-Participant Parents

Prior to each focus group, participants will be reminded that whilst the session will be recorded, and the researcher will take notes as needed, the recording will be destroyed following full data analysis They will also be reminded that everything they discuss will remain anonymous and they can contribute when they choose to do so. They will also be reminded that there are no right or wrong answers, that different people might have different views on the intervention, and we are interested in finding out everyone's experience of the intervention. They will then be given an opportunity to ask questions before the focus group begins.

Introductory Questions

These questions are intended to put the participants at ease and encourage them to discuss their experiences over the past few weeks whilst completing the study. They will focus on topics such as:

Who they are, and, general information about themselves.

Main Questions

1) What are your overall opinions of the study intervention that was proposed?
2) What did you think about your child and an older adult pairing up to work together?
   Good idea? Bad idea? Why? What did you child think?
3) Were you comfortable approaching the adult(s) about their potential participation?
   What stopped you asking? Why were you uncomfortable?
4) You all identified that your child in theory had an older adult of the right age who could have participated, why do you think they did not want to?
5) What are your thoughts /beliefs/opinions on older adults being physically active and exercising? Why?
6) Do you know how much exercise your child / older adults should be trying to do?
   Are you surprized? What do you think about these guidelines?
7) Do you have any thoughts or ideas on what we could do to improve uptake to similar studies or physical activity interventions?

<u>Summary</u>

The focus group will be concluded with a reminder that if participants want to withdraw their data from the study, they only need to contact the Researcher without offering a reason.

Participants will be thanked for their time and given the opportunity to ask questions about the study within the group or in private after the group.
