# Supplementary material for: Moving together: Increasing physical activity in older adults with an intergenerational technology-based intervention. A feasibility study
Source: PLoS One. 2024 Mar 27;19(3):e0301279. doi: 10.1371/journal.pone.0301279 (PMC10971589; doi:10.1371/journal.pone.0301279)
Supplement: S4 File — (PDF) [file pone.0301279.s004.pdf]

S4 File

Coding Audit Trail
Excerpts of Initial Manual Coding

Morgan:	Well I wanted to get involved to be honest um firstly for Jesse uh because she was she's really into keeping fit and environmental issues and all this type of thing if she can walk rather than ride she will um and because I enjoy knitting sewing and all the rest of it I took it as a challenge for me (.) which it has been um it's definitely shown me when I'm active and when I'm not um (.) I look after my granddaughter a little one two days a week sometimes three and then my steps are really down because I've got her and obviously most of the time is just spent running or trotting after her as opposed to going on long walks (.) Um but what I have tried to do, I've always enjoyed doing it when I've had the time but what this has made me do is (.) make time which has been a very positive thing for me, it's more of a fitness thing as opposed to a pleasure thing I must admit but I can definitely feel that I am that much fitter ((pause)) so for me it's served a purpose.

Challenge → engagement with + int. self-Monitoring Awareness of levels

Getting involved for someone else. PA Awareness. Time. Positive experience. +ve exp.

Viv:	Um (.) but he he easily beat me on most days there were two or three days when I beat him ((laughter)) um (.) basically I haven't done anything out of the ordinary other than I have found myself more aware of exercise um (.) like I walk around the bathroom cleaning my teeth now ((laughter)) and when vacuuming the carpet instead of standing on the spot and going like this ((demonstrates)) I go striding down the hallway and striding back up again ((laughter)) so its made me more conscious then
Francis:	((In overlap)) It does though doesn't it
Viv:	Of of walking and and finding ways of incorporating activity in the normal daily routine.
Pat:	I think I can say I found some of the same things as the previous two, um particularly I noticed looking after [grandchild] a 3 year old, step counts right down on those days and that's not because I'm not active it's just because I'm not out walking or gardening or doing other things (.) Um I didn't (.) do anything out of the ordinary as far as I'm aware perhaps the first couple of days I walked down to town instead of taking the car but the novelty of that soon wore off but um I do have some peaks if you look at the data on mine where um usually on a Thursday where my wife and I

Competition. ↑ PA Awareness. ↑ PA. ↑ PA Awareness. PA Awareness. Novelty Factor.

Example of Data Extraction

	Code	Data Extract
Usability	Parents wearing watches for children	<u>*Focus group 2 – Children*</u><br>*Jesse (p5):* My [parent] wears mine so [they] actually does the steps for me for a bit ((laughter))<br>*Alex (p8):* I go swimming for an hour, so I got my [parent] to wear the watch and then and then I got it to 12,000<br>*Casey (p7):* I see my [parent] opening the door when I come swimming [their] jogging on the spot
Usability	Compliance	<u>*Focus group 1 – Older Adults*</u><br>*Viv (p3):* But sometimes [they] went out to [place] and forgot to put [their] watch on<br><br><u>*Focus group 2 – Children*</u><br>*Case (p7)y*: Well I did leave it somewhere, but I tried to get more steps up (.)<br><br>*Jesse (p5):* My [parent] wears mine so [they] actually does the steps for me for a bit ((laughter))<br>*Alex (p8):* I go swimming for an hour so I got my [parent] to wear the watch and then and then I got it to 12,000<br>*Casey (p7):* I see my [parent] opening the door when I come swimming [their] jogging on the spot<br><br>*Casey (p7):* Have you ever left you fit bit somewhere like me<br><br>*Jesse (p5):* That’s mine I was trying really hard but I took it off for like 2 hours of the day<br>*Case (p7)y:* I’m still here even though I’ve left this in the toilet (laughter) and I left it in the (bed) cos it’s not waterproof that’s why I took it off
Usability	Technological limitations World Walking	<u>*Focus group 1 – Older Adults*</u><br>*Viv (p3):* ......there was such a long gap between er I was stuck in Portmerion for ever because there was no base at Aberystwyth or anywhere<br><br>*Pat (p2):* I think there was there are some issues with that where it stops at a certain point like a junction on a road and nothing seems to happen until you’ve done a certain number more steps and then suddenly it jumps forward<br><br>*Viv (p3):* My main gripe was the distance between Portmerion to Fishguard or St David’s<br><br>*Viv (p3):* Also, if your taking about thee um actual website there were medals that you were supposed to get (.) none of my medals were given<br>*Francis:* I had a walking the penguin one, march of the penguins or something<br>*Pat (p2):* Did you? I didn’t get any either<br>*Viv (p3):* And er on the first day if you remember when we were setting the watches Casey already clocked up the solo walk one and then we got another one of the symbols it clicked in and then after that nothing<br><br>*Viv (p3):* I check it every night and go <u>what</u> I haven’t moved I’m still in Portmerion<br><br>*Viv (p3):* Did you ever go on the street view on it as well?<br>*Pat (p2):* By accident<br>*Viv (p3):* If you clicked on street view you were sometimes stuck on a round about<br>*Francis:* or a bend in the road or something
Usability	Psychological / motivational limitations from activity monitor	<u>*Focus group 1 – Older Adults*</u><br>*Morgan: (p1)* Right right the other thing I found a bit unnerving on a poor day it would say (here) ahead of nine percent of users and I’d think oh god I’m in trouble<br><br>*Francis (p4):* I think Alex was disappointed because [they] do a lot of swimming and obviously it’s not waterproof<br><br>*Interviewer:* we could provide a sheet and there are some out there already that give calculations and we could give them some information on for example if you swam for

		thirty minutes you can allocate yourself 'x' amount of steps or a formula where if you did gymnastics for an hour allocate yourself um a certain number of steps<br>*Morgan (p1):* Yeah<br>*Viv (p3):* [Name] goes swimming as well<br>*Morgan (p1):* That would encourage them then I think they'd be more into it because Jesse often said oh I did this and I did that but I had to take my watch off<br><br>*<u>Focus group 2 – Children</u>*<br>*Alex (p8):* ((In overlap)) that's why I didn't do many steps<br>Interviewer: You would have liked one that you could have kept on in the water?<br>*Alex (p8):* Yes because I could have beated my [grandparent]
**Usability**	Technological limitations activity monitor	*<u>Focus group 1 – Older Adults</u>*<br>*Francis (p4):* [they] does a lot of swimming so a lot of [theirs] don't count<br><br>*Morgan (p1):* I did what I was finding was well there was one day where it just logged me out completely I don't know and then thankfully you logged me back in but um what I also found was that I'd go to bed at night check how much I'd done but the next morning it was different so what I started to do was I've got a stepper as well there was one day where (.) there we are it was in the second week on the Tuesday in the night (.) before I went to bed right I had done six thousand three hundred and fifty five the next morning I had done fifty-eight........ what I started doing was I'd take a note every night then of what I'd done and then that is the figure I would put on the sheet the next morning not what the watch shows what my phone shows<br><br>*Francis (p4):* I think Alex was disappointed because [they] do a lot of swimming and obviously it's not waterproof<br>*Morgan (p1):* Yes, Jesse found that and [they do] gymnastics as well and of course they can't keep wear them for gymnastics<br><br>*Pat (p2):* It didn't seem to matter what I did it never registered me as doing anything strenuous<br><br>*Morgan (p1):* I think the concept is positive I've had issues with my phone and the watch a little bit<br>*Francis (p4):* I couldn't wear it at night I didn't like it on my wrist at night<br><br>*<u>Focus group 2 - Children</u>*<br>*Alex (p8):* I'm actually getting one of my own which is waterproof<br>*Interviewer:* Ah<br>*Casey (p7):* I'd like one waterproof cos I always go swimming on a Thursday<br>*Jesse (p5):* I do, I go on a Tuesday<br>*Casey (p7):* I'm in wave 8<br>*Alex (p8):* ((In overlap)) I go 5 times a week<br>*Jesse (p5):* ((In overlap)) I'm on wave 5<br>*Alex (p8):* ((In overlap)) that's why I didn't do many steps<br><br>*Casey (p7):* I'm still here even though I've left this in the toilet (laughter) and I left it in the (bed) cos it's not waterproof that's why I took it off

Example of Sub-theme Refinement Process

Acceptability	Usability	Functionality	Recruitment & Retention	Additional
Engagement	***Technological Limitations***	***Participation Stimuli***	***Perceptions***	
Engagement lead to insight into / awareness of activity levels	Technological limitations Activity Monitor	Competition	Perceived time for PA as a potential recruitment limitation	Perceptions of PA & guidelines
Engagement did not lead to insight into / awareness of activity levels	Technological limitations World Walking	Collaboration	Perceived ability to use technology	Barriers to physical activity
Engagement with the activity monitor – additional features	Parents wearing watches for children	Importance of Individualisation & Goal Achievability	Perceptions of research as a recruitment limitation	Facilitators of physical activity
Engagement with the activity monitor – step counts	Compliance	Motivator	Perceived Technophobia	
Engagement with app features	***Psychological Limitations***		Perceptions of technology as a potential recruitment limitation	
Frequency of engagement with technology	Psychological / motivational limitations from Activity Monitor	***Outcomes***	Self-Perceptions of Ageing	
Engagement with World Walking / Maps	Psychological / motivational limitations from World Walking	Positive impact on PA levels	Views-on-ageing: older adults are set in their ways	
Conceptual Limitations	***Operationality***	Positive experience - Found time for physical activity	*Perception of the intervention concept	
Impact of Terminology	Self-Monitoring	**Incorporation into daily routine	***Mediators***	
Potential novelty factor	Ease of usage - positive	Changes to contact between the dyad	Mediating effect of parents on recruitment + Family dynamics as a recruitment limitation = *Family Dynamics*	
Positive Experience	Ease of usage - negative		(Lack of) Interest in physical activity	
Wider Benefit	Need for Parental Involvement		Information overload	
Positive engagement / experience with technology	How information was transferred between the dyad		Reason for Participation	
Participation provided an overall positive experience			(Lack of) Access to technology	
Potential for longer term participation			(Lack of) Interest in technology	
			Facilitators	
*Perception of the intervention concept			Recruitment via children	
**Incorporation into daily routine			Use of incentives in increase recruitment	
			Opinions on alternative partnership options	
			Generativity	

Acceptability	Usability	Functionality	Recruitment & Retention	Additional
Engagement	***Technological Limitations***	***Participation Stimuli***	***Perceptions***	
Engagement lead to insight into / awareness of activity levels	Technological limitations World Walking	***Motivator***	Perceived time for PA as a potential recruitment limitation	Perceptions of PA & guidelines
Engagement did not lead to insight into / awareness of activity levels	***Technological limitations Activity Monitor***	***Individualisation & Goal Achievability***	*Perception of the intervention concept	Barriers to physical activity
Engagement with World Walking / Maps	Parents wearing watches for children	Importance of Goal Achievability	Perceptions of research as a recruitment limitation	Facilitators of physical activity
Frequency of engagement with technology	Compliance	Importance of Individualisation & Goal Achievability	Perceptions of technology as a potential recruitment limitation	
Engagement with the activity monitor & app	***Psychological Limitations***	***Competition v's Collaboration***	***Age Stereotypes***	
Engagement with the activity monitor – additional features	Psychological / motivational limitations from Activity Monitor	Competition	Perceived ability to use technology	
Engagement with the activity monitor – step counts	Psychological / motivational limitations from World Walking	Collaboration	Perceived Technophobia	
Engagement with app features	***Operationality***	***Generated Outcomes***	Self-Perceptions of Ageing	
	Self-Monitoring	Positive impact on PA levels	Views-on-ageing: Older adults are set in their ways	
Conceptual Limitations	Ease of usage - positive	Positive experience - Found time for physical activity	***Mediators***	
Impact of Terminology	Ease of usage - negative	**Incorporation into daily routine	***Information overload***	
Potential novelty factor	Need for Parental Involvement	Changes to contact between the dyad	***Participatory Reasons***	
	How information was transferred between the dyad		Generativity	(Lack of) Access to technology? **should be removed only one vague reference to this**
Positive Experience	Frequency of engagement with technology		To be more active	
Wider Benefit			***Family Dynamics***	
Positive engagement / experience with technology			Mediating effect of parents on recruitment	
Participation provided an overall enjoyable experience			Family dynamics as a recruitment limitation	
Potential for longer term participation			***Level of interest***	
			Level of interest in physical activity	
*Perception of the intervention concept			Level of interest in technology	
**Incorporation into daily routine			***Facilitators***	
			Recruitment via children	
			Use of incentives	
			Potential alternative partnership options	

Final Coding by Theme, Sub-theme, and Codes

Framework Theme	Theme	Sub Theme	Code
**Acceptability**	**Engagement**	Insight	Engagement led to insight into / awareness of activity levels
			Engagement did not lead to insight into / awareness of activity levels
		Engagement with technology	Engagement with the activity monitor – additional features
			Engagement with the activity monitor – step counts
			Engagement with app features
			Engagement with World Walking / Maps
			Frequency of engagement with technology
**Acceptability**	**Positive Experience**		Wider Benefit
			Participation provided an overall enjoyable experience
			Positive engagement / experience with technology
			Potential for longer term engagement
**Usability**	**Limitations**	Technological limitations	Technological limitations World Walking
			Compliance
			Parents wearing watches for children
			Technological limitations from the activity monitor
		Psychological / Motivational limitations	Psychological / motivational limitations from Activity Monitor
			Psychological / motivational limitations from World Walking
**Usability**	**Operationality**	Ease of Usage	Ease of usage - negative
			Ease of usage - positive
			How information was transferred between the dyad
			Need for Parental Involvement
			Self-Monitoring

Framework Theme	Theme	Sub Theme	Code
**Functionality**	**Participant Stimuli**	Motivator	Motivator
		Individualisation	Importance of Individualisation
			Importance of Goal Achievability
		Competition versus Collaboration	Competition
			Collaboration
**Functionality**	**Generated Outcomes**		Positive experience - Found time for physical activity
			Changes to contact between the dyad
			Positive impact on PA levels
			Incorporation into daily routine
**Recruitment & Retention**	**Facilitators**		Recruitment via children
			Use of incentives
			Potential alternative partnership options
**Recruitment & Retention**	**Perceptions**	Perceptions of Ageing	Perceived ability to use technology
			Perceived Technophobia
			Self-Perceptions of Ageing
			Views-on-ageing: Older adults are set in their ways
			Perceptions of research as a recruitment limitation
			Perceived time for PA as a potential recruitment limitation
			Perceptions of technology as a potential recruitment limitation
			Perception of the intervention concept
**Recruitment & Retention**	**Mediators**	Family Dynamics	Family dynamics as a recruitment limitation
			Mediating effect of parents on recruitment
		Reasons for Participation	Reason for participation – To be more active
			Reason for participation - Generativity
		Level of Interest	Level of Interest in physical activity
			Level of interest in technology
		Information overload	Information overload
		Conceptual Limitations	Impact of Terminology

Final Coding Detailing Each Participant Groups Contribution to Each Theme

Frame work	Theme	Sub Theme	Code	Comments
**Acceptability**	**Engagement**	Insight	Engagement led to insight into / awareness of activity levels	<u>*Focus group 1 – Older Adults*</u><br>*Morgan:* .... it has been um it's definitely shown me when I'm active and when I'm not um (.)<br><br>*Viv:* .... I have found myself more aware of exercise um (.) like I walk around the bathroom cleaning my teeth now ((laughter)) and when vacuuming the carpet instead of standing on the spot and going like this ((demonstrates)) I go striding down the hallway and striding back up again ((laughter)) so it's made me more conscious then<br><br>*Francis:* ....it made me take more notice of what I was doing<br><br>*Morgan:* .... it's made me more aware of it (.) it made me more conscious of it and made me think and basically as you say rather than sit down and think aw I'll do something later I'll do it now<br><br>*Viv:* I think actually wearing the watch though makes you more conscious of it<br>Francis: Yeah I think oh I'll just pop up town now you know I won't get that tomorrow I'll get that now and then I'll up my steps today.<br><br>*Pat:* I noticed looking after [grandchild] a 3 year old, step counts right down on those days...... ....if you look at the data on mine where um usually on a Thursday where my [spouse] and I go to Penllegaer Valley Woods to volunteer and we're walking around there doing gardening moving stuff around, that really pushed my steps up<br><br>*Morgan:* .... it has been um it's definitely shown me when I'm active and when I'm not um (.) I look after my [grandchild] a little one two days a week sometimes three and <u>then</u> my steps are really down because I've got [them]<br><br><u>*Focus group 2 – Children*</u><br>*Casey:* Well I did leave it somewhere but I tried to get more steps up (.)<br><br>*Jesse:* I enjoyed it because like you um got to know how many steps you've done<br><br>*Jesse:* Uh but yeah but I think we should do it again because it might actually help people um be encouraged a bit more to do a bit more walking and get fit more
**Acceptability**	**Engagement**	Insight	Engagement did not lead to insight into / awareness of activity levels	<u>*Focus group 2 – Children*</u><br>*Interviewer:* So (.) by doing more steps did you feel that you were moving a little bit more than you normally do? Did it make you think that Ooo I haven't done any steps perhaps I should try and do a bit more?<br>((Shaking of heads))<br>*Interviewer:* No? you were just interested to see how many<br>*Alex:* ((In overlap)) You do
**Acceptability**	**Engagement**	Engagement with technology	Engagement with the activity monitor – additional features	<u>*Focus group 1 – Older Adults*</u><br>*Viv:* I'd I notice sometimes I would monitor sometimes my heart rate if I was just idling round the house doing it could be as low as sixty something and then if I was out in the garden digging or something it would be up to one hundred and twelve so obviously um yeah<br>*Francis:* I don't think mine was ever more than eighty-five when I have taken it<br>*Viv:* Really well mine mine swung up over a hundred a few times<br><br>Alex: I liked it because you got to count your steps and you knew your heartbeat<br><br>*Taylor:* I really liked it cos um you could see your heart rate and stuff<br><br>*Taylor:* I liked it cos I had a clock in the middle of the night but now it's hard to get back used to using my other clock<br>*Alex:* I liked it because I could see the time

**Acceptability**	**Engagement**	Engagement with technology	Engagement with the activity monitor – step counts	*<u>Focus group 1 – Older Adults</u>*<br>*Pat:* I noticed looking after [grandchild] a 3 year old, step counts right down on those days...... ....if you look at the data on mine where um usually on a Thursday where my [spouse] and I go to Penllegaer Valley Woods to volunteer and we're walking around there doing gardening moving stuff around, that really pushed my steps up<br><br>*Viv:* Yes yeah it was I was gardening<br>*Pat:* ((In overlap)) Yeah<br>*Viv:* that my steps were up<br><br>*Viv:* And uh back and fore pulling up the moss with the machine I really had quite a high count on that day<br><br>*Viv:* On the day that I um excelled myself and I did over 11,000 steps<br><br>*Pat:* I knew about the ten thousand step target that people set and thought that's not achievable really so I set mine at five thousand like you and I didn't achieve that everyday even though that's quite a low figure really<br>*Viv:* I know but if I was just under in it you know in the evening I'd walk around the lounge until I got it over it<br>*Francis:* I'd say that after tea I'm just popping around the block so that I get up to my eight thousand<br><br>*<u>Focus group 2 – Children</u>*<br>Casey: ......I tried to get more steps up (.)<br><br>Alex: I liked it because you got to count your steps<br><br>*Jesse:* I enjoyed it because like you um got to know how many steps you've done<br><br>*Taylor:* .....I liked it because I could know how many steps I was doing<br><br>*Alex:* I got I got to 12,000 the other day
**Acceptability**	**Engagement**	Engagement with technology	Engagement with app features	*<u>Focus group 1 – Older Adults</u>*<br>*Pat:* It didn't seem to matter what I did it never registered me as doing anything strenuous.<br>*Morgan:* It registered me as doing fast walking<br>*Pat:* Did it<br>*Viv:* Yes yes I got some fast walking if I was late going up to the school<br><br>*Viv:* Well did you monitor you heart rate when you were gardening say and things like that on the watch?<br>*Pat:* No well it wasn't really convenient to do that I was just looking at one of my err ((pause)) days working in the woods when I walked six point six four miles and burnt three hundred and eighteen calories that doesn't sound much does it<br><br>*Morgan*: Right right the other thing I found a bit unnerving on a poor day it would say (here) ahead of nine percent of users and I'd think oh god I'm in trouble......<br>I was quite pleased here (.) ahead of 63% of users that's more like it<br><br>*Pat:* I found it quite addictive using the watch actually<br>*Morgan:* Yes<br>*Pat:* Um not just for the steps but for the sleep patterns as well, we were having a chat about that earlier on um (.) I found that quite fascinating I'm tempted to buy one to keep it going<br><br>*Viv:* Yes (.) I found that interesting as well because um I felt that I sleep better than I thought and (.) I found that information very interesting even more interesting really than how many steps I'd taken I would say

**Acceptability**	**Engagement**	Engagement with technology	Engagement with World Walking / Maps	*Focus group 1 – Older Adults*<br><br>*Viv:* I'd phone up and tell [them] you can stick a sticker on Bangor<br><br>*Viv:* [their parent] would email me the steps in the evening and then I would add it on when I went to bed so um in a sense it was a little bit disjointed that it was [parent] giving me the information after [they'd] gone to bed but [parent] said I've got to do that [parent] cos sometimes [they] come back down the stairs and [they've] clocked up more steps<br><br>*Morgan:* It worked the other way with us um I'm not terribly er gadget orientated so I would either text or ring or my [person-in-law] would ring me and I'd give them the steps and they'd monitor it<br><br>*Pat:* I think the map was the main thing it was the interest really in seeing how far we were getting it was good for us and good for the children as well"<br>*Francis:* Yeah I think they liked the map didn't they<br><br>*Morgan:* Um I think [person]-in-law has been putting us together<br>*Francis:* Yeah<br>*Interviewer:* It will add them together when you put them in<br>*Francis:* We put ours on together either I would put them on or [they] and Alex would put them on<br><br>*Morgan:* ....my [person]-in-law is doing the world walking for us<br><br>*Morgan:* in my case anyway [name] my [person]-in-law [they were] on the phone most nights you know saying what are your steps because we were [they] was doing the round Wales one and uh [they were] saying we got as far as this and Jesse had the map and of course [they were] following it as well ...<br>*Viv:* Did you ever go on the street view on it as well<br>*Pat:* By accident<br>*Viv:* If you clicked on street view you were sometimes stuck on a round about<br>*Francis:* or a bend in the road or something<br><br>*Focus group 2 – Children*<br>*Jesse:* .....it's really interesting having like a fake walk around the earth<br><br>*Casey:* Ooo Ooo I liked it because I keep getting putting stickers on my umm map<br>*Alex:* I like it because of the stickers as well<br><br>*Taylor:* I like working I like doing it because cos cos if if I was doing it on my own I wouldn't have got very far but when we when me and my [grandparent] were working as a team we got quite far<br><br>*Interviewer:* Let's have a little look (.) wow did you all like using your maps<br>*Alex:* Yes<br>*Casey:* Yep<br>*Alex or Taylor:* I loved it<br>*Jesse:* Yeah yeah yeah yeah<br><br>*Interviewer:* So [your parent] was looking at [your grandparents] map and telling you then how far you had done<br>*Jesse:* Yeah
**Acceptability**	**Engagement**	Engagement with technology	Frequency of engagement with technology	*Focus group 1 – Older Adults*<br>*Viv:* I check it every night (World Walking)<br><br>*Pat:* what I started doing was I'd take a note every night then of what I'd done and then that is the figure I would put on the sheet the next morning not what the watch shows what my phone shows<br><br>*Viv:* [their parent] would email me the steps in the evening and then I would add it on when I went to bed

**Acceptability**	**Positive Experience**		Wider Benefit	*Focus group 2 – Children*<br>*Taylor:* Um I liked it because um I because I we got to do a lot more walks and now I get to know a few more birds because we've gone out for lots more walks<br><br>*Jesse:* Umm I really enjoyed having the um the watches cos erh I could now go um with runs with my [parent] and stuff<br><br>*Alex:* I would like to do it again because I can phone my [grandparent] more and [they come] over more cause [they] sometimes [they] normally just comes over for my birthday or something else
**Acceptability**	**Positive Experience**		Positive engagement / experience with technology	*Focus group 1 – Older Adults*<br>*Francis:* then I had the watch and I thought well this is really good<br><br>*Francis:* I found it quite addictive using the watch actually<br><br>*Pat:* I think the map was the main thing it was the interest really in seeing how far we were getting it was good for us and good for the children as well<br><br>*Focus group 2 – Children*<br>*Alex:* I liked it because you got to count your steps and you knew your heartbeat<br><br>*Jesse:* I enjoyed it because like you um got to know how many steps you've done and erh it's really interesting having like a fake walk around the earth<br><br>*Taylor:* I really liked it cos um you could see your heart rate and stuff I liked it because I could know how many steps I was doing<br><br>*Jesse:* Umm I really enjoyed having the um the watches cos erh I could now go um with runs with my [parent] and stuff<br>*Taylor:* I liked it cos I had a clock in the middle of the night but now it's hard to get back used to using my other clock<br>*Alex:* I liked it because I could see the time<br>*Casey:* I liked it because I don't really have a smart watch that I own<br><br>*Interviewer:* Fab, so you all liked having a watch is what I think I'm picking up off you?<br>*Alex:* Yeah<br>*Interviewer:* If you were able to have them for longer or you were able to gets ones of your own is that something that you'd like to do?<br>*Casey:* Yes<br>*Jesse:* Nodding

**Acceptability**	Positive Experience		Participation provided an overall positive experience	*Focus group 1 – Older Adults*<br>*Interviewer:* Okay so is there anything other than the fact that World Walking stuck a little bit that in general you disliked about the study as a whole or the intervention?<br>*Viv:* No<br>*Pat:* Dislike<br>*Viv:* I didn't dislike anything really<br><br>*Pat:* I thought it was good because um ((pause)) I think you have a different relationship with your grandchildren to your children to some extent and so it was although we see a lot of ours it was just a nice thing to do<br><br>*Interviewer:* So over all would you recommend this type of intervention (pause) to be carried on?<br>*Morgan:* Yes<br>*Viv:* Yes.........<br>..........*Francis:* Yeah I've enjoyed it<br>*Morgan:* Yeah<br>*Pat:* I think it's quite addictive in a way<br>*Francis:* It is isn't it<br><br>*Francis:* I think it's mainly been positive for me<br>*Morgan:* I think the concept is positive<br><br>*Pat:* Yeah Taylor rang me and [they don't] normally ring me and was quite chatty on the phone talking about this and it was quite nice from that point of view but we do see [them] regularly anyway so.<br><br>*Francis:* Yeah and I've quite enjoyed doing that and it made me feel fitter and better for doing it<br><br>*Focus group 2 – Children*<br>*Interviewer:* So (.) going on what Casey said about beating [their grandparent] (.) what did you all think about having to work with your [grandparents] I think it was wasn't it? What did you think about having to work with them?<br>*Jesse:* I really enjoyed it<br><br>*Taylor:* I like working I like doing it because cos cos if if I was doing it on my own I wouldn't have got very far but when we when me and my [grandparent] were working as a team we got quite far<br><br>*Interviewer:* Let's have a little look (.) wow did you all like using your maps<br>*Alex:* Yes<br>*Casey:* Yep<br>*Alex or Taylor:* I loved it<br>*Jesse:* Yeah yeah yeah yeah<br><br>*Interviewer:* We'll just go back to your grandparents a little bit, the person that you were working with<br>*Alex:* ((In overlap)) Yeah<br>*Interviewer:* did you like working with them<br>*Jesse:* Yeah I did<br>*Interviewer:* Do you think they liked that<br>*All Together:* Yes ((Nodding))<br>*Interviewer:* So how did it make you feel do you think taking part with them?<br>*Jesse:* Um I<br>*Casey:* Great<br>*Jesse:* I felt I felt really good

**Acceptability**	**Positive Experience**		Potential for longer term engagement	*<u>Focus group 1 – Older Adults</u>*<br>*Pat:* I found it quite addictive using the watch actually<br>*Morgan:* Yes<br>*Pat:* Um not just for the steps but for the sleep patterns as well, we were having a chat about that earlier on um (.) I found that quite fascinating I'm tempted to buy one to keep it going<br>*Francis:* Alex is going to try and get one that [they] can wear swimming because [they] really enjoyed doing it and [they] want one that [they] can wear in the water see how much activity [they do] then cos [their] quite interested<br><br>*Morgan:* when this trial is over we are going to carry on the four of us no five four Jesse Mum Dad me and target walking to the moon<br><br>*Interviewer:* Is it something that you would consider carrying on with? Well I already know the answer for one person (.) but is it something that you would consider continuing on with?<br>*Viv:* Yes<br>*Pat:* Yeah, I think so I'm seriously considering it<br>*Viv:* I'm considering buying<br>*Viv:* Yeah so I would consider buying one because it does make you feel like ow you know I'll<br>*Morgan:* ((In Overlap)) I need to do a bit more now<br><br>*<u>Focus group 2 – Children</u>*<br>*Alex:* I'm actually getting one of my own which is waterproof
**Usability**	**Limitations**	Technological limitations	Technological limitations World Walking	*<u>Focus group 1 – Older Adults</u>*<br>*Viv:* ......there was such a long gap between er I was stuck in Portmerion for ever because there was no base at Aberystwyth or anywhere<br><br>*Pat:* I think there was there are some issues with that where it stops at a certain point like a junction on a road and nothing seems to happen until you've done a certain number more steps and then suddenly it jumps forward<br><br>*Viv:* My main gripe was the distance between Portmerion to Fishguard or St David's<br><br>*Viv:* Also if your taking about thee um actual website there were medals that you were supposed to get (.) none of my medals were given<br>*Francis:* I had a walking the penguin one, march of the penguins or something<br>*Pat:* Did you? I didn't get any either<br>*Viv:* And er on the first day if you remember when we were setting the watches Casey already clocked up the solo walk one and then we got another one of the symbols it clicked in and then after that nothing<br><br>*Viv:* I check it every night and go <u>what</u> I haven't moved I'm still in Portmerion<br><br>*Viv:* Did you ever go on the street view on it as well?<br>*Pat:* By accident<br>*Viv:* If you clicked on street view you were sometimes stuck on a round about<br>*Francis:* or a bend in the road or something

**Usability**	**Limitations**	Technological limitations	Compliance	<u>*Focus group 1 – Older Adults*</u><br>*Viv:* But sometimes [they] went out to [place] and forgot to put [their] watch on<br><br><u>*Focus group 2 – Children*</u><br>*Casey*: Well I did leave it somewhere but I tried to get more steps up (.)<br><br>*Jesse:* My [parent] wears mine so [they] actually does the steps for me for a bit ((laughter))<br>*Alex:* I go swimming for an hour so I got my [parent] to wear the watch and then and then I got it to 12,000<br>*Casey:* I see my [parent] opening the door when I come swimming [their] jogging on the spot<br><br>*Casey:* Have you ever left you fit bit somewhere like me<br><br>*Jesse:* That's mine I was trying really hard but I took it off for like 2 hours of the day<br>*Casey:* I'm still here even though I've left this in the toilet (laughter) and I left it in the (bed) cos it's not waterproof that's why I took it off
**Usability**	**Limitations**	Technological limitations	Parents wearing watches for children	<u>*Focus group 2 – Children*</u><br>*Jesse:* My [parent] wears mine so [they] actually does the steps for me for a bit ((laughter))<br>*Alex:* I go swimming for an hour so I got my [parent] to wear the watch and then and then I got it to 12,000<br>*Casey:* I see my [parent] opening the door when I come swimming [their] jogging on the spot
**Usability**	**Limitations**	Technological limitations	Technological limitations from the activity monitor	<u>*Focus group 1 – Older Adults*</u><br>*Francis:* [they] does a lot of swimming so a lot of [theirs] don't count<br><br>*Morgan:* I did what I was finding was well there was one day where it just logged me out completely I don't know and then thankfully you logged me back in but um what I also found was that I'd go to bed at night check how much I'd done but the next morning it was different so what I started to do was I've got a stepper as well there was one day where (.) there we are it was in the second week on the Tuesday in the night (.) before I went to bed right I had done six thousand three hundred and fifty five the next morning I had done fifty-eight........ what I started doing was I'd take a note every night then of what I'd done and then that is the figure I would put on the sheet the next morning not what the watch shows what my phone shows<br><br>*Francis:* I think Alex was disappointed because [they] do a lot of swimming and obviously it's not waterproof<br>*Morgan:* Yes Jesse found that and [they do] gymnastics as well and of course they can't keep wear them for gymnastics<br><br>*Pat:* It didn't seem to matter what I did it never registered me as doing anything strenuous<br><br>*Morgan:* I think the concept is positive I've had issues with my phone and the watch a little bit<br>*Francis:* I couldn't wear it at night I didn't like it on my wrist at night<br><br><u>*Focus group 2 - Children*</u><br>*Alex:* I'm actually getting one of my own which is waterproof<br>*Interviewer:* Ah<br>*Casey:* I'd like one waterproof cos I always go swimming on a Thursday<br>*Jesse:* I do, I go on a Tuesday<br>*Casey:* I'm in wave 8<br>*Alex:* ((In overlap)) I go 5 times a week<br>*Jesse:* ((In overlap)) I'm on wave 5<br>*Alex:* ((In overlap)) that's why I didn't do many steps<br><br>*Casey:* I'm still here even though I've left this in the toilet (laughter) and I left it in the (bed) cos it's not waterproof that's why I took it off

**Usability**	**Limitations**	Psychological / Motivational limitations	Psychological / motivational limitations from Activity Monitor	<u>*Focus group 1 – Older Adults*</u><br>*Morgan:* Right right the other thing I found a bit unnerving on a poor day it would say (here) ahead of nine percent of users and I'd think oh god I'm in trouble<br><br>*Francis:* I think Alex was disappointed because [they] do a lot of swimming and obviously it's not waterproof<br><br>*Interviewer:* we could provide a sheet and there are some out there already that give calculations and we could give them some information on for example if you swam for thirty minutes you can allocate yourself 'x' amount of steps or a formula where if you did gymnastics for an hour allocate yourself um a certain number of steps<br>*Morgan:* Yeah<br>*Viv:* Casey goes swimming as well<br>*Morgan:* That would encourage them then I think they'd be more into it because Jesse often said oh I did this and I did that but I had to take my watch off<br><br><u>*Focus group 2 – Children*</u><br>*Alex:* ((In overlap)) that's why I didn't do many steps<br>Interviewer: You would have liked one that you could have kept on in the water?<br>*Alex:* Yes because I could have beated my [grandparent]
**Usability**	**Limitations**	Psychological / Motivational limitations	Psychological / motivational limitations from World Walking	<u>*Focus group 1 – Older Adults*</u><br>*Viv:* .....I thought it would motivate me to get the bike out the back of the garage and go on a cycle ride with Casey you see but no it was all about walking<br><br>*Viv:* ......there was such a long gap between er I was stuck in Portmerion for ever because there was no base at Aberystwyth or anywhere (.) and that kind of<br>*Morgan:* There was nothing in between<br>*Viv:* Yeah there was nothing in between<br>*Morgan:* It disheartens you a bit<br>*Viv:* It did yes there should have been a<br>*Francis:* If you go on you feel the next milestone is St David's don't you and you think your never gonna get there<br><br>*Pat:* .....it was just a bit irritating to think you'd done a couple of days, quite a lot of steps and you hadn't got anywhere<br><br>*Viv:* I check it every night and go <u>what</u> I haven't moved I'm still in Portmerion<br><br>*Viv:* to know that you're at Aberystwyth but it's not a ((pause))<br>Interviewer: It wasn't a milestone<br>*Pat:* Milestone yeah<br>*Viv:* Milestone that's the word and that was <u>dee</u> motivating<br><br>*Pat:* I also thought it would be nice if it was slightly shorter so that there was a chance of getting to the end<br><br>*Viv:* .....when we were setting the watches Casey already clocked up the solo walk one and then we got another one of the symbols it clicked in and then after that nothing so that was kind of a little bit demotivating as well<br>*Pat:* I didn't understand what that was all about to be perfectly honest<br>*Viv:* I know I looked at them and I think it was because some of them might be some of them were probably linked to particular walks like a mountain climbing or something<br>*Interviewer:* I'll check<br>*Viv:* But none of those lit up again and I thought oh we can't be making very good progress

**Usability**	**Operationality**	Ease of Usage	Ease of usage - negative	*Focus group 1 – Older Adults*<br>*Pat*: .....I found it a bit baffling the day we came in and you explained it all to us but once I you know you explained it well we set things up together I went home and read through the guidelines and then it was okay after that I didn't have any problems at all to be honest<br>Viv: No I didn't have any problems either<br>*Francis:* Once it was sort of set up I was okay (.) I couldn't set it up<br><br>*Morgan:* I did what I was finding was well there was one day where it just logged me out completely I don't know and then thankfully you logged me back in but um what I also found was that I'd go to bed at night check how much I'd done but the next morning it was different so what I started to do was I've got a stepper as well there was one day where (.) there we are it was in the second week on the Tuesday in the night (.) before I went to bed right I had done six thousand three hundred and fifty five the next morning I had done fifty-eight........ what I started doing was I'd take a note every night then of what I'd done and then that is the figure I would put on the sheet the next morning not what the watch shows what my phone shows
**Usability**	**Operationality**	Ease of Usage	Ease of usage - positive	*Focus group 2 – Children*<br>*Interviewer:* Okay, were the watches difficult to use or did you find them quite easy<br>*Taylor:* Easy<br>*Alex:* Very easy<br>*Casey:* Quite easy<br>*Interviewe*r: Did you have any problems with them<br>*Alex:* No
**Usability**	**Operationality**		How information was transferred between the dyad	*Focus group 1 – Older Adults*<br>*Viv:* I'd phone up and tell [them] you can stick a sticker on Bangor<br><br>*Pat:* Taylor used to ring me I didn't ring [them] by the way<br><br>*Viv:* [their parent] would email me the steps in the evening and then I would add it<br><br>*Morgan:* I would either text or ring or my [person-in-law] would ring me and I'd give them the steps and they'd monitor it<br><br>*Alex:* if I spoke to Alex on the phone and they'd say 'how many steps you done?' and if I'd done more than them they'd tell me 'talk to [parent]<br><br>*Pat:* ....my [person]-in-law [they were] on the phone most nights you know saying what are your steps because we were [they] was doing the round Wales one and uh [they were] saying we got as far as this and Jesse had the map and of course [they were] following it as well and saying 'come on [grandparent] you need to do more'....<br><br>*Focus group 2 – Children*<br>*Taylor:* I I'd sometimes ring my [grandparent] or my [grandparent] would get me to text [them] on [their] phone and [they'd] tell me like I'd done that many steps and tell us where we'd got to<br><br>*Casey:* .....my [grandparent] picks me up so I I already know what [they've] done<br>*Interviewer:* So [they'd] tell you when [they] picked you up?<br>*Casey:* Yes<br><br>*Jesse:* Um, I knew how far I'd got it's because my [parent] could have a look on [their] phone cos [they] could see how far we'd got on [their] phone<br>*Interviewer:* So [your parent] was looking at [your grandparents] map and telling you then how far you had done<br>*Jesse:* Yeah

**Usability**	**Operationality**		Need for Parental Involvement	<u>*Focus group 1 – Older Adults*</u><br>*Morgan:* my [person]-in-law [they were] on the phone most nights you know saying what are your steps<br><br>*Viv:* ......[their parent] would email me the steps in the evening and then I would add it on when I went to bed so um in a sense it was a little bit disjointed that it was mum giving me the information after he'd gone to bed but she said I've got to go that [parent] cos sometimes [they] come back down the stairs and [they've] clocked up more steps<br><br>*Morgan:* I'm not terribly er gadget orientated so I would either text or ring or my [person]-in-law would ring me and I'd give them the steps and they'd monitor it<br><br>*Morgan:* Um I think [person]-in-law has been putting us together<br><br>*Francis:* We put ours on together either I would put them on or [they] and Alex would<br><br>*Morgan:* my [person]-in-law is doing the World Walking for us<br><br><u>*Focus group 2 – Children*</u><br>*Jesse:* Um, I knew how far I'd got it's because my [parent] could have a look on [their] phone cos [they] could see how far we'd got on [their] phone<br>*Interviewer:* So [your parent] was looking at [your grandparents] map and telling you then how far you had done<br>*Jesse:* Yeah<br><br>*Casey:* My [parent] didn't need to look at my smart watch because I'm just upstairs and in the corner my [parent] can just umm sync it in
**Usability**	**Operationality**		Self-Monitoring	<u>*Focus group 1 – Older Adult*</u><br>*Morgan:* ....it's definitely shown me when I'm active and when I'm not um (.) I look after my [grandchild] a little one two days a week sometimes three and <u>then</u> my steps are really down<br><br>*Viv:* I really had quite a high count on that day<br><br>*Pat:* ......we're walking around there doing gardening moving stuff around, that really pushed my steps up which you know I found quite satisfying<br><br>*Francis:* Yeah I think oh I'll just pop up town now you know I won't get that tomorrow I'll get that now and then I'll up my steps today<br><br>*Pat:* Or if you have a day when you don't do much like when you're looking after one of the grandchildren you think oh tomorrow I've gotta do some extra<br><br>*Pat:* I knew about the ten thousand step target that people set and thought that's not achievable really so I set mine at five thousand like you and I didn't achieve that everyday even though that's quite a low figure really<br>*Viv:* I know but if I was just under in it you know in the evening I'd walk around the lounge until I got it over it<br><br>*Francis:* I'd say that after tea I'm just popping around the block so that I get up to my eight thousand<br><br>*Pat:* That's why I set mine at five because I thought well that's a bit of a challenge for me to to do that every day<br><br><u>*Focus group 2 – Children*</u><br>*Alex:* I liked it because you got to count your steps<br><br>*Jesse:* I enjoyed it because like you um got to know how many steps you've done and erh it's really interesting having like a fake walk around the earth<br><br>*Taylor:* I really liked it cos um you could see your heart rate and stuff I liked it because I could know how many steps I was doing<br><br>*Alex:* I got I got to 12,000 the other day

Frame work	Theme	Sub Theme	Code	Comments
**Functionality**	**Participant Stimuli**	Motivator	Motivator	*Focus group 1 – Older Adults*<br>*Viv:* Well I felt sort of motivated by the fact that I was you know measuring how much activity I was taking<br><br>*Pat:* that really pushed my steps up which you know I found quite satisfying<br><br>*Pat:* I found it quite addictive using the watch actually<br><br>*Francis:* then I had the watch and I thought well this is really good and then you get like a bit competitive with yourself don't you<br>*Viv:* ((In overlap)) Yeah<br>*Francis:* and you think ahh I'll do a bit more tomorrow and that's how it works<br><br>*Morgan:* in my case anyway [name] my [person]-in-law [they were] on the phone most nights you know saying what are your steps because we were [they] was doing the round Wales one and uh [they were] saying we got as far as this and Jesse had the map and of course [they were] following it as well and saying 'come on [grandparent] you need to do more'<br><br>*Morgan:* ......it's good in that way in that it's made me more aware of it (.) it made me more conscious of it and made me think and basically as you say rather than sit down and think aw I'll do something later I'll do it now you know so it does it's a good<br>*Viv:* Motivator<br>*Morgan:* It is a good motivator there's no doubt about that, for me anyway<br><br>*Morgan*: And I think you said the purpose is to measure the steps and all the rest of it and to motivate people it's definitely done that for me<br><br>*Francis:* Whereas before I used to think oh I don't know now I'll get up and go for a walk you know what I mean rather than lie in bed and watch the news ((Laughter)) and have a cup of tea but I've got up and gone for a walk and then come back and watched the news and had a cup of tea you know<br>*Viv:* So it does motivate us more<br><br>*Viv:* Well I think it was motivating.<br><br>*Francis:* ......they'd say walk a bit faster [grandparent] I want to get to St David's<br><br>*Focus group 2 – Children*<br>*Jesse:* I enjoyed it because like you um got to know how many steps you've done and erh it's really interesting having like a fake walk around the earth
**Functionality**	**Participant Stimuli**	Individualisation	Importance of Individualisation	*Focus group 1 – Older Adults*<br>*Francis:* It puts it at eight thousand anyway doesn't it so you feel like an achievement<br>*Viv:* It depends where you set your target my target was only five thousand<br><br>*Pat:* I knew about the ten thousand step target that people set and thought that's not achievable really so I set mine at five thousand like you and I didn't achieve that everyday even though that's quite a low figure really<br><br>*Pat:* I think the important thing is if you do think about setting targets for people is they have got to be achievable otherwise you get that demotivating factor coming in<br>*Viv:* Yes if you'd set ours at ten thousand steps a day I don't think ….<br>*Pat:* Yeah we wouldn't have bothered<br><br>*Pat:* That's why I set mine at five because I thought well that's a bit of a challenge for me to to do that everyday<br><br>*Pat:* Because of the things we've been talking about but I thought it was achievable and I think I averaged about seven thousand anyway my higher days compensated for the lower ones

**Functionality**	**Participant Stimuli**	Individualisation	Importance of Goal Achievability	<u>*Focus group 1 – Older Adults*</u><br>*Morgan:* I didn't realise that you need to do you know 10,000 steps is not far off 5 miles a day for me which is quite a lot to do you know when they say you should be doing 10,000 steps a day there's no way I could do that I don't think<br><br>*Morgan:* But I must admit I am disappointed that you know 10,000 steps is pretty much 5 miles that's a fair amount to do in a day I don't think I couldn't do that every day I don't think<br><br>*Francis:* It puts it at eight thousand anyway doesn't it so you feel like an achievement<br><br>*Interviewer:* ......we didn't set you a goal ((Pause)) quite interestingly you all set yourselves goals. Did that help? Would you have helped if we'd set you goals? Or did you prefer that you had that freedom to set yourself something and change it as you saw fit?<br>*Viv:* That would depend on how high you'd set our goal ((Laughter))<br>*Morgan:* I would say from my point of view that um that would demotivate me for the simple reason that looking after grandchildren looking after a little toddler you know on those days I know full well I'm not going to do that type of step number of steps (.)<br><br>*Pat:* I think the important thing is if you do think about setting targets for people is they have got to be achievable otherwise you get that demotivating factor coming in<br>*Viv:* Yes if you'd set ours at ten thousand steps a day I don't think ….<br>*Pat:* Yeah we wouldn't have bothered<br>((Laughter))<br>*Pat:* That's why I set mine at five because I thought well that's a bit of a challenge for me to do that everyday.

**Functionality**	**Participant Stimuli**	Competition versus Collaboration	Competition	<u>*Focus group 1 – Older Adults*</u><br>*Viv:* [they they] easily beat me on most days there were two or three days when I beat [them]<br><br>*Pat:* ......I found quite satisfying cos that was the only days that I managed to beat Taylor on steps<br><br>*Francis:* you get like a bit competitive with yourself don't you<br><br>*Viv:* I disliked that my [grandchild] can do more steps than I can do<br><br>*Morgan:* Jesse is quite competitive so yeah it did start off and it's been all the way through a sort of competition between us<br><br>*Francis:* It does make you more competitive I think I mean obviously if I spoke to Alex on the phone and they'd say 'how many steps you done?' and if I'd done more than them they'd tell me 'talk to [parent]'<br><br>*Interviewer:* .....we when I was setting it up we were quite and I was quite specific that I was trying to find something that where the task was collaborative and even though we've found a task that's collaborative it actually still appears and correct me if I'm wrong that it it's turned out quite competitive?<br>*Morgan:* Yes it was for us<br>*Francis:* Um um<br>*Interviewer:* Was that a problem?<br>*Morgan:* No<br>*Francis:* No<br>*Viv:* No<br>*Pat:* I wouldn't say it it er ((pause)) when we say was competitive it was just a bit of fun isn't it really.<br>*Francis:* ((In overlap)) Yeah um it was nicely done<br><br>*Morgan:* ......I was quite pleased here (.) ahead of 63% of users that's more like it<br>*Pat:* Who says we're not competitive<br><br>*Viv:* Well [they] usually beat me<br><br><u>*Focus group 2 – Children*</u><br>*Casey:* I like it because I keep beating my [grandparent]<br><br>*Alex:* My [grandparent] kept beating me because [they] had to walk up and down to my [relative] because [they live] in a different (.) because [they live] in England and they were on holiday and then so she walked there and then she forgot something and then she had to walk all the way back. So that's how [they] beat me every day.<br>*Casey:* My [grandparents] very slow ((laughter)) as I keep beating [them]<br><br>*Interviewer:* Other than Casey you thought (.) were you competing with your [grandparent] or did you think you were working as a team with your [grandparent]<br>*Casey:* No ((laughter))<br>*Interviewer:* No you weren't a team<br>*Casey:* No! ((shaking head))<br>*Interviewer:* You wanted to beat your [grandparent]<br>*Casey:* ((Nodding))<br>*Alex:* All the time<br><br>*Interviewer:* You would have liked one that you could have kept on in the water<br>*Alex:* Yes because I could have beated my [grandparent]

**Functionality**	**Participant Stimuli**	Competition versus Collaboration	Collaboration	*Focus group 1 – Older Adults*<br>*Morgan:* Jesse had the map and of course [they were] following it as well and saying 'come on [grandparent] you need to do more'....<br><br>*Francis:* they'd say walk a bit faster [grandparent] I want to get to St David's<br><br>*Pat:* I think the map was the main thing it was the interest really in seeing how far we were getting it was good for us and good for the children as well<br><br>*Focus group 2 – Children*<br>*Taylor:* ..... if I was doing it on my own I wouldn't have got very far but when we when me and my [grandparent] were working as a team we got quite far.<br>*Jesse:* Its um with me and my [grandparent] we got really far on the map<br><br>*Taylor:* I felt kind of happy because sometimes some days when we didn't go out for a walk I'd only do something like 2,000 when my [grandparent] would be out doing lots of steps so [they] kinda helped me when [they] didn't do lots of steps I did when I didn't do lots of steps [they] helped me do lots of steps
**Functionality**	**Generated Outcomes**		Positive experience - Found time for physical activity	*Focus group 1 – Older Adults*<br>*Morgan:* I've always enjoyed doing it when I've had the time but what this has made me do is (.) make time<br><br>*Pat:* But you don't always have the time do you that's the thing.<br>*Morgan:* What I found it has made me do is find the time<br>*Pat:* Right good<br>*Francis:* Yeah it does, doesn't it<br><br>*Francis:* "And I've enjoyed making the time as well"<br>*Morgan:* That's right<br>*Francis:* Whereas before I used to think oh I don't know now I'll get up and go for a walk you know what I mean rather than lie in bed and watch the news ((Laughter)) and have a cup of tea but I've got up and gone for a walk and then come back and watched the news and had a cup of tea you know

**Functionality**	Generated Outcomes		Changes to contact between the dyad	<u>*Focus group 1 – Older Adults*</u><br><br>*Morgan:* For me no because I see them quite often anyway<br>Viv: Yeah I see them nearly every day anyway apart from Saturdays and Sundays so just saw him the same.<br>*Francis:* I think I talked to Alex more<br>*Pat:* Yeah Taylor rang me and [they don't] normally ring me and was quite chatty on the phone talking about this and it was quite nice from that point of view but we do see [them] regularly anyway so<br><br><u>*Focus group 2 – Children*</u><br>*Taylor:* I I'd sometimes ring my [grandparent] or my [grandparent] would get me to text [them] on [their] phone and [they'd] tell me like I'd done that many steps and tell us where we'd got to<br>*Casey:* I got (.) my [grandparent] picks me up so I I already know what [they've] done<br>*Interviewer:* So [they'd] tell you when [they] picked you up?<br>*Casey:* Yes<br><br>*Alex:* Yes (.) yeah (.) so I phoned [them] more than I usually do.<br>*Interviewer:* So did you all speak to your grandmas and grandpas more than you normally do<br>*All Together:* Yes<br>*Casey:* Well not all the time<br>*Interviewer:* Do you think they liked that?<br>*All Together:* Yes ((Nodding))<br><br>*Interviewer:* Did any of you do any extra activities with your grandparents so that you both got steps together?<br>*Alex:* Uh ((puts up hand))<br><br>*Casey:* Every time when I come home my [grandparent] we'd run up the park and then we do a little jog around the top of there and come back to my [grandparents] and sometimes I go back to my house<br><br>*Alex:* I would like to do it again because I can phone my [grandparent] more and [they come] over more

**Functionality**	Generated Outcomes		Positive impact on PA levels	*<u>Focus group 1 – Older Adults</u>*<br>*Morgan:* .....I can definitely feel that I am that much fitter ((pause)) so for me it's served a purpose.<br><br>*Francis:* .... then I had the watch and I thought well this is really good and then you get like a bit competitive with yourself don't you<br>*Viv:* ((In overlap)) Yeah<br>*Francis:* and you think ahh I'll do a bit more tomorrow and that's how it works<br><br>*Francis:* ...you tend to instead of like you say like standing around doing things you tend to wander more and don't mind going up and down stairs and you don't just leave it on the stairs and go up later you go up and down with it<br><br>*Morgan:* ....rather than sit down and think aw I'll do something later I'll do it now you know<br><br>*Viv:* sometimes if it was a nice day I would walk the long way around I'd come right up to the [Club] and come around to the school that way which backfired one morning because they had locked the gate couldn't get in there was a crowd there so I just sort of circled round to clock up some steps<br><br>*Francis:* instead of driving to the supermarket I walk, cos I can like walk to the supermarket<br><br>*Francis:* I think oh I'll just pop up town now you know I won't get that tomorrow I'll get that now and then I'll up my steps today<br>*Viv:* Yeah so I would consider buying one because it does make you feel like ow you know I'll<br>*Morgan:* ((In Overlap)) I need to do a bit more now<br><br>*Francis:* Whereas before I used to think oh I don't know now I'll get up and go for a walk you know what I mean rather than lie in bed and watch the news ((Laughter)) and have a cup of tea but I've got up and gone for a walk and then come back and watched the news and had a cup of tea you know<br><br>*Viv:* I know but if I was just under in it you know in the evening I'd walk around the lounge until I got it over it<br>*Francis:* I'd say that after tea I'm just popping around the block so that I get up to my eight thousand<br><br>*<u>Focus group 2 – Children</u>*<br>Casey: Well I did leave it somewhere but I tried to get more steps up (.)<br><br>*Taylor:* Um I liked it because um I because I we got to do a lot more walks<br><br>*Alex:* Um I went um on a walk to around the common a lot<br>*Interviewer:* Which you wouldn't normally do?<br>*Alex:* No I would never do that unless I would do other things<br><br>*Jesse:* .....having these watches it actually is um helping me go to walking and running a lot a lot more<br><br>*Casey:* I like it because I can always wake up in the morning and have a little walk around the house and in the night my [parent] says oh go then run around the house<br><br>*Jesse:* Well my [grandparents] actually been walking around more than [they] usually does and [they've] started going for walks in the morning around [place] where [they live]

**Functionality**	**Generated Outcomes**		Incorporation into daily routine	*Focus group 1 – Older Adults*<br>*Viv:* .....I walk around the bathroom cleaning my teeth now ((laughter)) and when vacuuming the carpet instead of standing on the spot and going like this ((demonstrates)) I go striding down the hallway and striding back up again((laughter)) so it's made me more conscious then<br>*Francis:* ((In overlap)) It does though doesn't it<br>*Viv:* Of of walking and and finding ways of incorporating activity in the normal daily routine.<br><br>*Pat:* Um I didn't (.) do anything out of the ordinary as far as I'm aware perhaps the first couple of days I walked down to town instead of taking the car but the novelty of that soon wore off but um I do have some peaks if you look at the data on mine where um usually on a Thursday where my [spouse] and I go to [place] to volunteer and we're walking around there doing gardening moving stuff around<br><br>*Viv:* It was I was gardening ….that my steps were up…. Yeah when I scarified the lawn<br><br>*Francis:* ......you tend to instead of like you say like standing around doing things you tend to wander more and don't mind going up and down stairs and you don't just leave it on the stairs and go up later you go up and down with it<br><br>*Morgan:* ......basically as you say rather than sit down and think aw I'll do something later I'll do it now<br><br>*Viv:* ......I got some fast walking if I was late going up to the school<br>((Laughter))<br>*Viv:* and sometimes if it was a nice day I would walk the long way around I'd come right up to the [Club] and come around to the school that way which backfired one morning because they had locked the gate couldn't get in, there was a crowd there so I just sort of circled round to clock up some steps<br><br>*Francis:* I think instead of driving to the supermarket I walk cos I can like walk to the supermarket<br>*Morgan:* ((In Overlap)) I would park in town and then walk around and do the shopping<br>*Francis:* Yeah I think oh I'll just pop up town now you know I won't get that tomorrow I'll get that now and then I'll up my steps today
**Recruitment & Retention**	**Facilitators**		Recruitment via children	*Focus group 1 – Older Adults*<br>*Morgan:* Right I'd have thought a lot of it would've come from the children um I'm in it because Jesse wanted to do it you know if she had come home and said oh they've got this thing or this letter and I don't really wanna do it mum and I don't really wanna do it Dad then that would be the end of it.<br>*Interviewer:* So your suggesting that we need to sell it<br>*Morgan:* sell it to the children<br><br>*Morgan:* Because they would then go home and encourage each other as well I think<br>*Francis:* Once one is doing it it makes other ones want to get involved<br><br>*Morgan:* .... regardless of technology a lot comes from the children Jesse was desperate to start because Taylor had one<br>*Pat:* Really ((Laughter))<br>*Morgan:* Yeah yeah I think it comes from the children
**Recruitment & Retention**	**Facilitators**		Use of incentives	*Focus group 3 – Decliners*<br>*Sam:* A couple of honestly a couple if a couple of um surveys I've gone you get ten pounds for them each<br><br>*Sam:* Or cinema tickets for them for the participants<br><br>*Blake:* Whatever their movement you could offer you know like a free gym session<br>*Charlie:* An incentive<br>*Blake:* Like an incentive whether it's a free like dance session<br>*Charlie:* A book a book<br>*Blake:* You could do dance three times a week for free you can do this you can get a swimming pass for the month, you can go swimming instead you know if that's gonna get you moving we will help you get you moving

**Recruitment & Retention**	Facilitators		Potential alternative partnership options	<u>*Focus group 1 – Older Adults*</u><br>*Interviewer:* we could contemplate making it rather than a dyad a triad and actually having the parent involved ....<br>*Viv:* I don't think it would have influenced how I felt about it but of course it would have depended on whether the parent was working or not working.<br>*Morgan:* Yeah Yep and the type of jobs that they've got<br>*Viv:* If they've got a sedentary job sitting at a computer all day then you haven't got as much opportunity to be getting up and walking around.<br><br>*Interviewer:* So it's not so much thinking about how many steps or how fit they would be to participate in it its whether do you think that dynamic would change if rather than you had that pairing of you and you grandchild if we introduced other family members and made it more of a family group rather than a child and grandparent group<br>*Morgan:* I think it would be good but I think you'd miss out because my [child] and [person]-in-law are quite active um as you say but if some parents sit down all day then ((pause)) I don't think that would work to be honest.<br><br>*Pat:* I don't know why it wouldn't work obviously every family is different<br><br>*Pat:* Yeah um if you're looking at motivating people to do more exercise then I don't think it would do any harm to have extra you know to have the parents involved<br>*Morgan:* I think the less mobile ones perhaps<br>*Viv*: Yeah they might get up and walk from the computer to the photocopier a bit more often<br><br>*Morgan:* As I say mine are quite fit .....their very conscious anyway so I don't think that particular family would benefit that much from it whereas because its Jesse and me and we're competing then I think that's a better motivator if that's what your after is motivation<br><br><u>*Focus group 3 – Decliners*</u><br>*Interviewer:*.......so tell me a little bit more about what you thought about your child and an older adult pairing up to work together regardless of what we've talked about as in whether they knew each other or not (.) but in general what do you think about that concept?<br><br>*Sam:* Yeah fine my older boy goes to rugby and there's older coaches there which they sometimes have close contact so yeah I have no problem.<br><br>*Sam:* If it was the parent and the child I think you would have had more people doing it<br>*Charlie:* I agree there definitely<br>*Interviewer:* So if we'd had the parent the child (.) if we'd allowed the parent to be involved as well do you think that might have encouraged your older adult to be involved as well?<br>*Sam:* No<br>*Charlie:* No ((Laughter))<br>*Interviewer:* If they'd had you to deal with all of the technology?<br>*Blake:* Maybe if we'd then said you just literally have to wear it and we'll do all of the datas<br><br>*Blake:* I think if mine was involved I'd be happy with [child] being matched with another adult if you had other adults who wanted to take part and if I was in you know (.)
**Recruitment & Retention**	Perceptions	Perceptions of Ageing	Perceived ability to use technology	<u>*Focus group 1 – Older Adults*</u><br>*Viv:* I would actually wonder about if it's the technology that put older people off a little bit because not everyone over sixty-five is conversant with modern technology

**Recruitment & Retention**	Perceptions	Perceptions of Ageing	Perceived Technophobia	<u>*Focus group 1 – Older Adults*</u><br>*Viv:* I would actually wonder about if it's the technology that put older people off a little bit because not everyone over sixty-five is conversant with modern technology<br>*Francis:* That's true<br>*Viv:* We're not afraid of doing things online and all the rest of it but some people um who may not have been in that type of employment say......<br><br><u>*Focus group 3 – Decliners*</u><br>*Blake:* ....I think it's that their old and technophobes<br>*Charlie:* I think that's what it is as well<br>*Blake:* I'd already bought my [other parent] like a smart watch to try and encourage [them] to move more and I think it just sits in a draw so I think maybe technology is (.)<br>*Charlie:* Yeah it's that age as well<br>*Sam:* Yeah<br>*Blake:* Yeah it's often<br>*Charlie:* I think they just about cope with a mobile phone don't they<br><br>*Blake:* I don't know how to get over the technophobe bit at the end of the day I think that's what put definitely my [in-law] but also my [parent] lots of odd excuses<br><br>*Blake:* um and then just technology really I think you know people you know I don't think you'd get over that mindset with some old people and the elderly<br><br>*Sam:* ....and saying that [their] a technophobe farmer so obviously we're saying it's yeah that their so set in their ways
**Recruitment & Retention**	Perceptions	Perceptions of Ageing	Self-Perceptions of Ageing	<u>*Focus group 3 – Decliners*</u><br>*Blake:* My [parent] has been to lots of funerals lately of people [they] used to work with and now for them it's like life's to short we're living our life now we're seventy something if we go tomorrow we've had a good time sort of thing I was like yeah but you could have another twenty thirty years<br><br>*Blake:* Yeah they often you know know that they need to be doing more but then their often in the mindset like with mine well my [parent] goes 'well I'm seventy-two' I'm not going to lose weight now am I I'm not going to and I'm like well you could actually you could get fitter you could move more but [they are] you know 'I've had a good life' and 'I'm seventy something' 'I'm gonna keep as I'm going'

**Recruitment & Retention**	**Perceptions**	Perceptions of Ageing	Views-on-ageing: Older adults are set in their ways	<u>*Focus group 3 – Decliners*</u><br>*Blake:* ......they're so set in routines that<br>*Charlie:* Yeah<br>*Blake:* The joke is that they used to moan about my [grandparent] being set in [their] routine but as they get older their turning into [them]<br>*Charlie:* Yeah they do yeah it is and I think it's an age thing once cos they get to a certain age they have their routine you know and it doesn't matter what you do you will not change their routine<br><br>*Charlie:* I think it's as well an age thing as well they're all set in their own ways of what they will do at certain times and they've got routines and I think that is what is the main problem from my aspect<br><br>*Charlie:* I think it's a generation thing<br><br>*Blake:* Yeah but you know I think just for my lot it literally just well it was going to interfere with their day to day life having to wear a watch and I was like it's <u>not</u> but they're so in a routine<br><br>*Charlie:* ((In overlap)) Well they do go out but they do the same thing every week<br><br>*Blake:* The dog is always walked at the same time<br>*Charlie:* ((In overlap)) Teatime is a certain time you have dinner tea it's all at the same time<br>*Blake:* When they come to ours it's like "do you want a cup of tea?" and my [parent] will look at [their] watch and rather than go 'yeah I'm quite thirsty actually I'll have a cup of tea' [they] look at [their] watch it's like well<br>*Charlie:* It's not teatime yet I can't have a cup of tea it's not eleven o'clock<br>*Blake:* If someone says to me "do you want a cuppa tea?" I'm like yeah I'm gasping thanks I'd love a cup of tea thanks<br>*Charlie:* It's not eleven o'clock you can't have one<br><br>*Charlie:* Yeah it is yeah set days they do certain things shopping days<br><br>*Blake:* you've got to try surely (.) I don't yeah obviously I'm not in their I'm not in their heads but I think that's what it is with them their not gonna you know change<br>*Sam:* That's the problem with your age group is that they're stubborn<br>*Charlie:* Yeah it's an age thing<br>*Blake:* I just don't want to turn out like them<br><br>*Interviewer:* But what you but you seem to be implying that you think we need to start targeting change in<br>*Sam:* People's perceptions and activities before they get to that age<br>*Charlie:* Yeah yeah<br>*Sam:* That's the problem with your age group is that they're stubborn<br>*Charlie:* Yeah it's an age thing<br>*Sam:* Yeah<br>*Charlie:* It is and I think that's why I think if you made it that bit younger perhaps you can get<br>people because they'll think you know I think I would say anything from over fifty perhaps because they are more active anyway and I dun know

**Recruitment & Retention**	**Perceptions**		Perceptions of research as a recruitment limitation	<u>*Focus group 3 – Decliners*</u><br>*Charlie:* It's just that personally I didn't have anyone who I knew would be happy to participate in things like this<br><br>*Charlie:* .....I just know that he wouldn't be interested I just think I dun know and like my in-laws they don't move enough to do it<br>*Blake:* My [in-law] was instantly um "what I've got to wear something?" and we were like 'yeah it's a watch'… and "but I wear a watch already" I was like 'yes' …. "what it tracks me?"<br><br>*Charlie:* Yeah perhaps if it wasn't the meeting side of it and stuff I don't know whether that would have been better<br>*Sam:* Yeah just send it give us your address and we'll send it out to you and maybe a little pamphlet about how to start it<br>*Blake:* that you just wear it for four weeks and whenever in was and then send it back<br>*Sam:* and then just send it back after four weeks<br>*Blake:* I don't know about yeah whether the meeting thing was I dun know might have been<br><br>*Sam:* And some old people "will you take part in this study?" "oh no no" straight away (.) I think that's what<br>*Charlie:* I think it's a generation thing<br>*Sam:* Yeah will you take part no no<br>*Charlie:* It's like when they used to knock the door saying will you do a survey they'd be like nah<br><br>*Blake:* ....maybe it is that if they are you know an older generation they just haven't had that contact to know that a study or a research study isn't anything invasive it's not I don't (.)<br>*Sam:* Whether they are going to steel all your information and stuff<br>*Blake:* Yeah or "what will the watch tell them? And is it going to take my heart rate?" and you know I don't know<br>*Sam:* "Will it say where I've been going?" little things like that innit<br>*Blake:* Yeah yeah little things maybe that just go through their mind
**Recruitment & Retention**	**Perceptions**		Perceived time for PA as a potential recruitment limitation	<u>*Focus group 3 – Decliners*</u><br>*Sam:* They've just got too much too much on<br><br>*Charlie:* ....I was going to ask anyone it was going to be my [parent] and he's always doing this doing that I just know that he wouldn't be<br><br>*Charlie:* I think it's as well an age thing as well they're all set in their own ways of what they will do at certain times and they've got routines and I think that is what is the main problem from my aspect<br><br>*Blake:* I'd like to you know I really like to say to them well why? Why wouldn't you but as you say if they're so set in routines that<br><br>*Blake:* The joke is that they used to moan about my [grandparent] being set in [their] routine but as they get older their turning into [them]<br>*Charlie:* Yeah they do yeah it is and I think it's an age thing once cos they get to a certain age they have their routine you know and it doesn't matter what you do you will not change their routine<br><br>*Charlie:* I don't think [they] would have done it because [their] too busy because [they've] always got stuff to do (.)[they are] quite active for [their] age [they are] active you know<br><br>*Blake:* Yeah but you know I think just for my lot it literally just well it was going to interfere with their day to day life having to wear a watch and I was like it's <u>not</u> but they're so in a routine
**Recruitment & Retention**	**Perceptions**		Perceptions of technology as a potential recruitment limitation	<u>*Focus group 3 – Decliners*</u><br><br>*Blake:* My [in-law] was instantly um "what I've got to wear something?" and we were like 'yeah it's a watch'… and "but I wear a watch already" I was like 'yes' …. "what it tracks me?" and then my [spouse] just gave up and were like it's fine [their] fit [their] sixty-five but [they] was like "no no no" and [their] an active [person] but it would still have been good for [them] and for [child]<br><br>*Blake:* Yeah or "what will the watch tell them? And is it going to take my heart rate?" and you know I don't know<br>*Sam:* "Will it say where I've been going?" little things like that innit<br>*Blake:* Yeah yeah little things maybe that just go through their mind (.) we need to bring them in there we go

**Recruitment & Retention**	Perceptions		Perception of the intervention concept	<u>*Focus group 3 – Decliners*</u><br>*Blake:* I thought it was lovely I had children who wanted to take part and were keen to take part so (.) yeah<br><br>*Charlie:* Yeah um I thought it was good it just that I knew that the older the age of the older participant I knew they wouldn't be happy to participate but my child was very keen<br><br>*Blake:* I think it's a lovely idea<br>*Charlie:* It's just that personally I didn't have anyone who I knew would be happy to participate in things like this as well<br><br>*Blake:* My [in-law] was instantly um "what I've got to wear something?" and we were like 'yeah it's a watch'… and "but I wear a watch already" I was like 'yes' …. "what it tracks me?"<br><br>*Charlie:* and [spouses' other parent] is just not fit enough it would be pointless<br><br>*Blake:* I think it was you know the idea would have been lovely I would have liked to have seen if I'd got some of my elderly relatives involved that they would have got moving more the you know that it would be my [child] ringing them sort of saying you know 'I've done eight thousand steps today [grandparent]'<br>*Charlie:* 'How many have you done?'<br>*Sam:* ((Overlap Laughter))<br>*Blake:* 'I beat you nahnahnahnahnah' as [they] would have done and it would have been nice to see my [parent] then get moving
**Recruitment & Retention**	Mediators	Family Dynamics	Family dynamics as a recruitment limitation	<u>*Focus group 1 – Older Adults*</u><br>*Pat:* I think I'm not quite sure what age you were looking at was eight no seven to eleven<br>*Interviewer:* Yes seven to eleven<br>*Pat:* So thinking about generations and going up twenty five years per generation roughly on average<br>*Morgan:* A lot of grandparents are younger than us<br>*Pat:* So yeah if you got early so people having children earlier in life you're not going to get a grandparent in that right bracket<br><br><u>*Focus group 3 – Decliners*</u><br>*Charlie:* Yeah um I thought it was good it just that I knew that the older the age of the older participant I knew they wouldn't be happy to participate but my child was very keen<br><br>*Blake:* I think it's obviously different family dynamics you have people that have had children in their twenties ....<br><br>*Blake:* You have people like me who've had children I'm obviously thirty to thirty-five my parents didn't have me until they were like thirty so they're in their seventies now I'm forty so you know<br>*Charlie:* ((In overlap)) Yeah that's right<br>*Blake:* So I think yeah it's just different family dynamics isn't it but um ((Pause)) I don't know if you'd get more<br><br>*Charlie:* I haven't got any older family really their all younger<br>*Sam:* Yeah I haven't got many I've got ten aunties and uncles who I never see so<br>*Charlie:* Yeah I've got loads of aunties and uncles but they're not in that age their all younger their all fifties.
**Recruitment & Retention**	Mediators	Family Dynamics	Mediating effect of parents on recruitment	<u>*Focus group 3 – Decliners*</u><br>*Charlie:* I knew that the older the age of the older participant I knew they wouldn't be happy to participate but my child was very keen<br><br>*Interviewer:* ......did you ask your parents to participate<br>*Charlie:* No<br>*Sam:* No<br><br>*Blake:* And they see obviously me and [my spouse] wear them and you just yeah [they] couldn't wait to wear one but

**Recruitment & Retention**	**Mediators**	Reasons for Participation	Reason for participation – To be more active	<u>*Focus group 1 – Older Adults*</u><br>*Morgan:* Well I wanted to get involved to be honest um firstly for Jesse ....and because I enjoy knitting sewing and all the rest of it I took it as a challenge for me<br><br>*Morgan:* Right I'd have thought a lot of it would've come from the children um I'm in it because Jesse wanted to do it you know if she had come home and said oh they've got this thing or this letter and I don't really wanna do it mum and I don't really wanna do it Dad then that would be the end of it<br><br><u>*Focus group 3 – Decliners*</u><br><br>*Blake:* ......I hoped that if they had participated they would have moved more and it would encourage them maybe to move more
**Recruitment & Retention**	**Mediators**	Reasons for Participation	Reason for participation - Generativity	<u>*Focus group 1 – Older Adults*</u><br>*Morgan:* Well I wanted to get involved to be honest um firstly for Jesse<br><br>*Morgan:* Right I'd have thought a lot of it would've come from the children um I'm in it because Jesse wanted to do it you know if she had come home and said oh they've got this thing or this letter and I don't really wanna do it mum and I don't really wanna do it Dad then that would be the end of it<br><br><u>*Focus group 2 – Children*</u><br>*Taylor:* I felt kind of happy because sometimes some days when we didn't go out for a walk I'd only do something like 2,000 when my [grandparent] would be out doing lots of steps so [they] kinda helped me when [they] didn't do lots of steps I did when I didn't do lots of steps [they] helped me do lots of steps<br><br><u>*Focus group 3 – Decliners*</u><br>*Blake:* I think that if you had have sold it on a that it's going to help their grandchildren do it on a then I dun know<br><br>*Blake:* I think sell you know you could have sold it to my lot in a come on you'd be really helping [child] this is for [child]<br><br>*Blake:* I hope that they'd see that it's not just helping them it's helping their grandchild
**Recruitment & Retention**	**Mediators**	Level of Interest	Level of Interest in physical activity	<u>*Focus group 3 – Decliners*</u><br>*Interviewer:* So why do you think [child] wasn't interested?<br>*Sam:* [Their] not [they] don't like doing anything [their] a typical teenager even though [their] eleven but [they are] a teenager [they'll] sit there watching TV or read read read<br><br>*Sam:* Yeah some of them just don't want to do it you know what I mean<br>*Charlie:* Yeah their all different you've got some kids which are really active and you've got other kids that aren't you know what I mean<br><br>*Blake:* I think the children have different characters you've got the ones who are going to be interested [Child] was chomping at the bit to have a go

**Recruitment & Retention**	Mediators	Level of Interest	Level of interest in technology	<u>*Focus group 1 – Older Adults*</u><br>*Morgan:* I'm rubbish with this type of thing and yet I was a [job] working with computers all day but anything to do with phones or anything like that and I'm rubbish You know? I'm not interested.<br>*Viv:* No But you've done computer work so you're not afraid of it?<br>*Morgan:* I'm not afraid of it but my [person]-in-law is doing the world walking for us<br><br>*Interviewer:* So just out of interest (.) your parents (.) so one of the things we talked about (.) technology (.) do they all have smart phones?<br>*Sam:* Yeah my [parent] doesn't use it though [they] doesn't [their] useless<br>*Blake:* My [parent] has a phone but my [other parent] doesn't well [they] do but it's normally switched off in [their place]<br>*Sam:* [My spouses] parents have phones but their just phones and just them my [parent] does have a smart phone<br><br><u>*Focus group 3 – Decliners*</u><br>*Interviewer:* do you think technology has the same sort of influence on the older generations now as well that they are using it more which<br>*Charlie:* They do use it more but I wouldn't say it has the same influence on the older generation as the younger generation<br>((Pause))<br>*Blake:* I suppose it's a man and women thing as well because my mum doesn't use a smart phone or that kind of thing she can get on my Dads phone and open up his Facebook page to see what people are doing she's nosey like that but she doesn't do online shopping<br>*Sam:* No<br>*Charlie:* Oh no<br>*Blake:* My dad will but she won't<br>*Charlie:* Yeah my Dad does but [spouses] parents don't
**Recruitment & Retention**	Mediators	Information overload	Information overload	<u>*Focus group 3 – Decliners*</u><br>*Sam:* Or maybe if you did a questionnaire to start with (.) what would you do or would you like to participate in and do this you know just quick short statements to show what the studies about and if they would be happy to do it<br><br>*Interviewer:* Almost like a poster (pause) or something with some information on maybe some pictures of what the<br><br>*Sam:* yeah cos it was a big folder wasn't it bit scary wasn't it you know what I mean<br><br>*Blake:* But then is it information overload for some elderly people you know<br><br>*Blake:* I have to say that when I emailed (.) obviously I asked you if you had an email copy that I could email to my [parent] and then [they] went "I've printed it off it's a lot of stuff"<br><br>*Sam:* .....((In overlap)) Yeah and it puts people off
**Recruitment & Retention**	Mediators	Conceptual Limitations	Impact of Terminology	<u>*Focus group 1 – Older Adults*</u><br>*Morgan:* I didn't realise that you need to do you know 10,000 steps is not far off 5 miles a day for me which is quite a lot to do you know when they say you should be doing 10,000 steps a day there's no way I could do that I don't think<br><br><u>*Focus group 3 – Decliners*</u><br>*Blake:* My [in-law] was instantly um "what I've got to wear something?" and we were like 'yeah it's a watch'… and "but I wear a watch already" I was like 'yes' …. "what it tracks me?"<br><br>*Blake:* Yeah or "what will the watch tell them? And is it going to take my heart rate?" and you know I don't know<br>*Sam:* "Will it say where I've been going?" little things like that innit<br>*Blake:* Yeah yeah little things maybe that just go through their mind (.) we need to bring them in there we go

**Recruitment & Retention**	**Mediators**	Conceptual Limitations	Potential novelty factor	<u>*Focus group 1 – Older Adults*</u><br>*Pat:* the first couple of days I walked down to town instead of taking the car but the novelty of that soon wore off<br><br>*Interviewer:* tell me a little bit more about did it encourage you to be more active?<br>*Morgan:* Yes<br>*Viv:* Yes definitely at the start yeah I would say its waned a little bit<br><br><u>*Focus group 2 – Children*</u><br>*Taylor:* I liked it cos I had a clock in the middle of the night but now it's hard to get back used to using my other clock<br>*Alex:* I liked it because I could see the time<br>*Casey:* I liked it because I don't really have a smart watch that I own<br><br>*Interviewer:* So (.) by doing more steps did you feel that you were moving a little bit more than you normally do? Did it make you think that Ooo I haven't done any steps perhaps I should try and do a bit more?<br>((Shaking of heads))<br>*Interviewer:* No? you were just interested to see how many<br>*Alex:* ((In overlap)) You do
